# Supplementary material for: The chromatin remodeller ATRX facilitates diverse nuclear processes, in a stochastic manner, in both heterochromatin and euchromatin
Source: Nat Commun. 2022 Jun 17;13:3485. doi: 10.1038/s41467-022-31194-7 (PMC9203812; doi:10.1038/s41467-022-31194-7)
Supplement: Supplementary file 1 — Supplementary Information [file 41467_2022_31194_MOESM1_ESM.pdf]

Supplementary Information

The chromatin remodeller ATRX facilitates diverse nuclear processes, in a stochastic manner, in both heterochromatin and euchromatin

Truch J., Downes D.J, Scott C., Gur E.R., Telenius J.M., Repapi E., Schwessinger R., Gosden M., Brown J.M., Taylor S., Cheong P.L., Hughes J.R., Higgs D.R. & Gibbons R.J.

Supplementary Figures

Supplementary Figure 1
Improved ATRX ChIP-seq protocol resulted in the production of sensitive and reproducible datasets in LCLs derived from three independent unaffected individuals and using two anti-ATRX antibodies.
Supplementary Figure 2
Genome-wide analysis of the ATRX binding sites confirmed ATRX affinity for repetitive regions, KRAB domains and loci containing C2H2 ZNF motif.
Supplementary Figure 3
Overlap analysis of random fragments matching in size and number the ATRX fragments under the same conditions.
Supplementary Figure 4
Validation of the Bio-CAP-seq in LCLs.
Supplementary Figure 5
Analysis of the subclasses of ATRX binding sites in LCLs based on the GenoSTAN annotation.
Supplementary Figure 6
Analysis of TFBS and motifs at ATRX binding sites.
Supplementary Figure 7
Differentiation of $CD34^+$ HSPCs.
Supplementary Figure 8
ATRX enrichment in cultured erythroblasts.
Supplementary Figure 9
Experimental design for the microarray analysis.
Supplementary Figure 10
ATRX mutations are associated with change in gene expression.
Supplementary Figure 11
Pathogenic ATRX mutations associated with changes in the chromatin environment.
Supplementary Figure 12
Southern blots assessing the methylation status at the promoter region of *ATF7IP2* and *ZNF555.*
Supplementary Figure 13
Chromatin interaction at the *PBX4* locus.
Supplementary Figure 14
Capture-C analysis at the alpha-globin cluster.
Supplementary Figure 15
scATAC-seq analysis of erythroblasts from ATR-X cases.
Supplementary Figure 16
Single cell analysis of erythroblasts from ATR-X case and unaffected donor.
Supplementary Figure 17
Information regarding flow cytometry.

Supplementary Tables

Supplementary Table 1
Gene ontology analysis based on the microarray data analysis from LCLs, ATR-X cases vs unaffected donors.
Supplementary Table 2
List of TaqMan® Assays from ThermoFisher Scientific.
Supplementary Table 3
List of primers used for qPCR.
Supplementary Table 4
Summary of the experimental replicates for high-throughput sequencing data and associated comments.
Supplementary Table 5
List of Capture-C oligos

a)

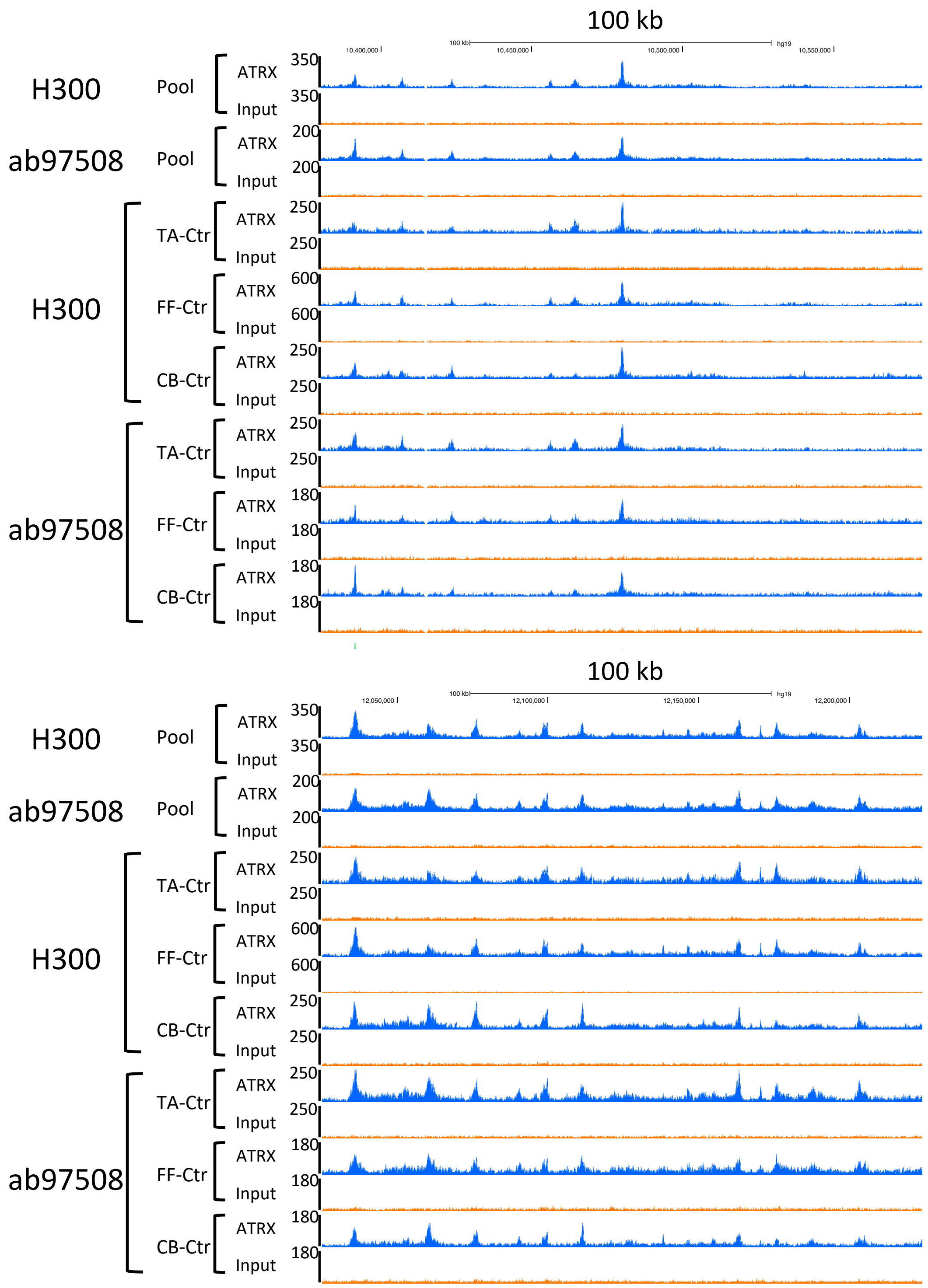
100 kb
H300
Pool
ATRX
Input
350
350
ab97508
Pool
ATRX
Input
200
200
H300
TA-Ctr
FF-Ctr
CB-Ctr
ATRX
Input
250
600
ab97508
TA-Ctr
FF-Ctr
CB-Ctr
180
100 kb

b)

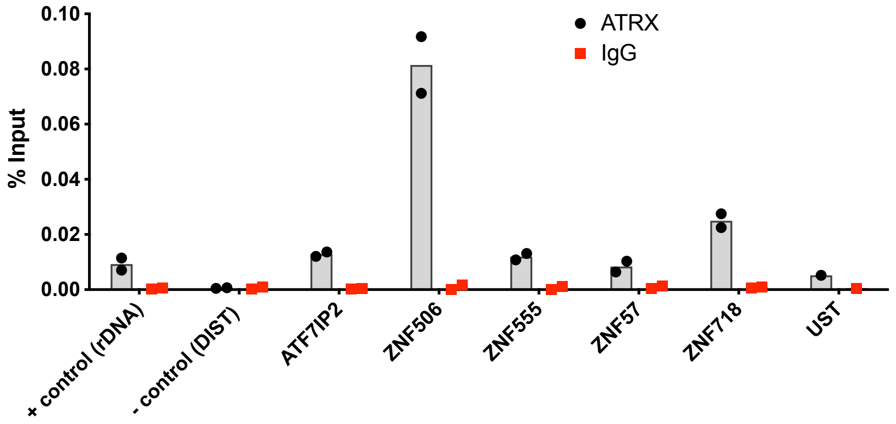
% Input
0.10
0.08
0.06
0.04
0.02
0.00
ATRX
IgG
+ control (rDNA)
- control (DIST)
ATF7IP2
ZNF506
ZNF555
ZNF57
ZNF718
UST

Supplementary Figure 1

Improved ATRX ChIP-seq protocol resulted in the production of sensitive and reproducible datasets in LCLs derived from three independent unaffected individuals and using two anti-ATRX antibodies (H300 and Ab97508) (n=1). a. Examples of 200kb regions with different types of ATRX enrichment. Even at regions highly enriched in ATRX (bottom panel), the associated inputs retain a very low background. Pool: pool of the three ATRX ChIP-seq experiments done on FF-Ctr, TA-Ctr and CB-Ctr LCLs using the same anti-ATRX antibody. H300: anti-ATRX antibody from Santa Cruz. Ab97508: anti-ATRX antibody from Abcam. b. Validation of ATRX enrichment by ChIP-qPCR at a subset of targets and using IgG ChIP-qPCR as a negative control (n=2 except for UST for which n=1, all data points are shown). + control: rDNA, - control: DIST. Source data are provided as a Source Data file.

a)

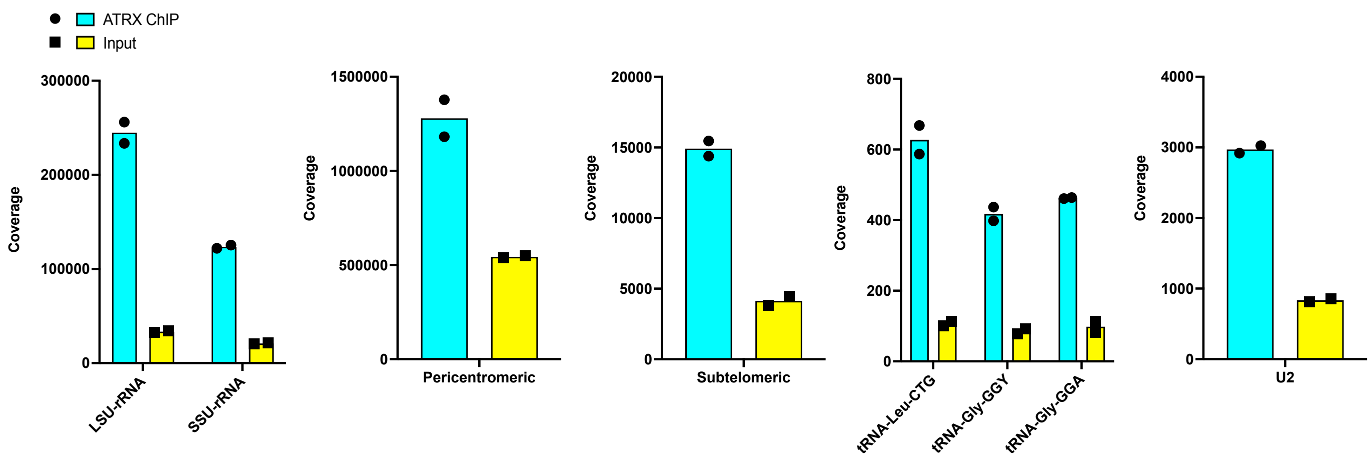

b)

Name	Overlap (number of peaks)	p-value
**GC_rich|Low_complexity|Low_complexity**	810	2,51E-28
**LTR2B|LTR|ERV1**	61	3,3E-21
**MER57E3|LTR|ERV1**	63	7,06E-17
**TGn|Simple_repeat**	425	1,55E-08
**CAn|Simple_repeat**	395	1,7E-08
**Simple_repeat**	1978	3,05E-07
**CGGn|Simple_repeat**	147	5,88E-07
**CCGn|Simple_repeat**	140	7,62E-07

c)

p-value	Term	Number of Genes in Term	Number of Target Genes in Term
**1.34E-86**	KRAB	350	253
**7.18E-76**	Znf_C2H2	766	404

d)

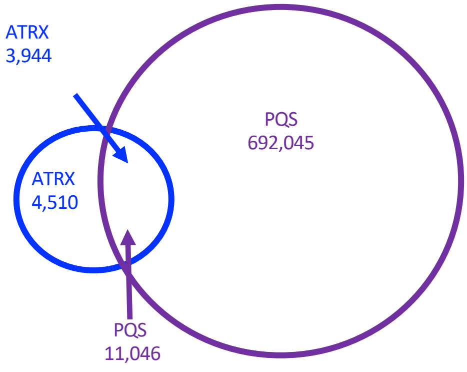

Supplementary Figure 2

Genome-wide analysis of the ATRX binding sites confirmed ATRX affinity for repetitive regions, KRAB domains and loci containing C2H2 ZNF motif. a. Direct mapping of ATRX ChIP-seq data to the repeatome highlighting the signal enrichment of ATRX over input at the long (LSU) and short (SSU) subunit of the rDNA gene locus, at pericentromeric and subtelomeric DNA, at a subset of genes encoding tRNA and at the small nuclear RNA (U2) loci (n=2, all data points are shown). b-c. Top terms of (b) the enrichment analysis at repetitive sequences, (c) the genome ontology. p-values HOMER annotatePeaks.pl (Heinz et al., 2010). d Venn diagram showing overlaps between ATRX binding sites and PQS genome-wide in LCLs.

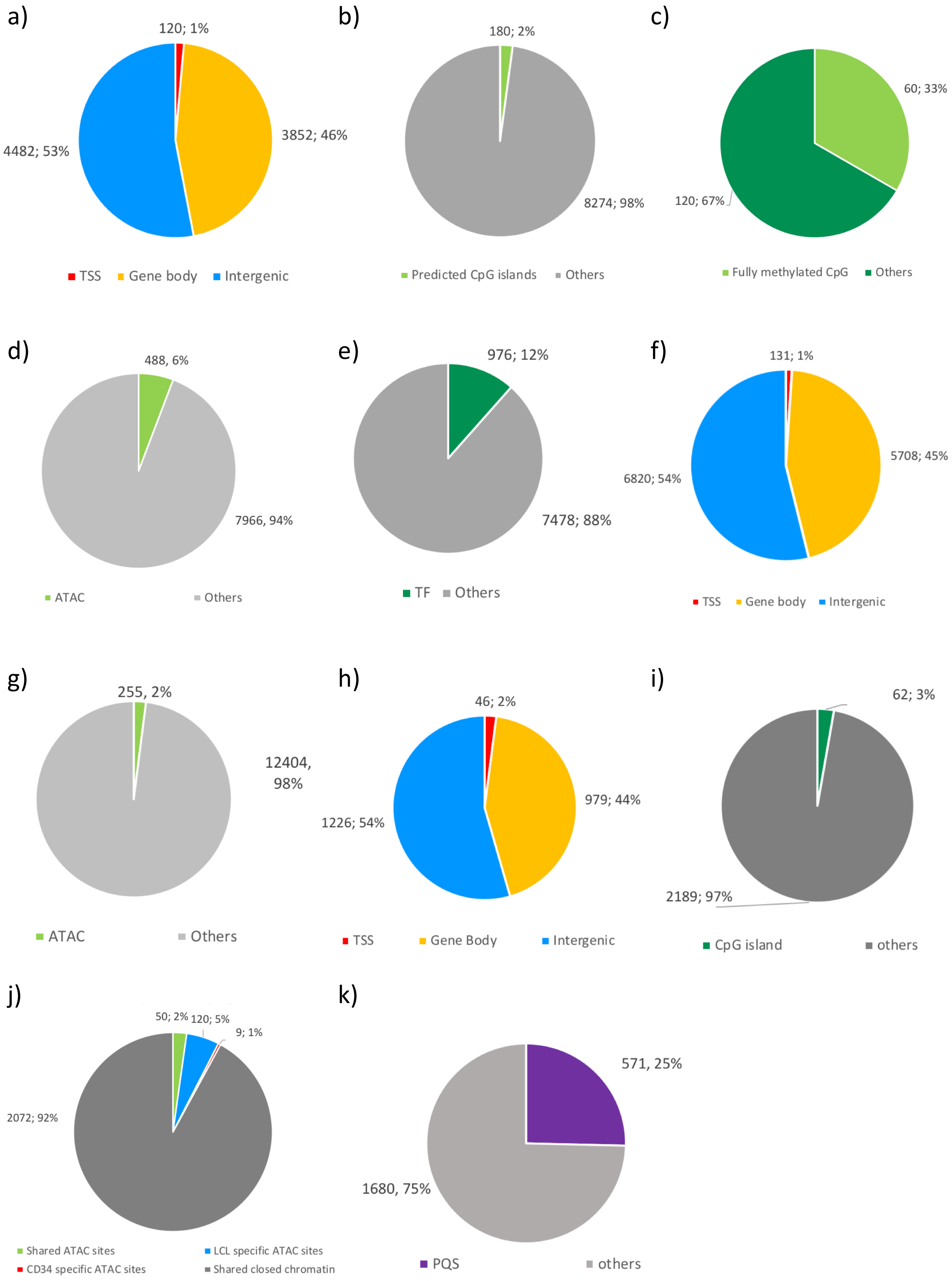

a)
120; 1%
3852; 46%
4482; 53%
TSS
Gene body
Intergenic
b)
180; 2%
8274; 98%
Predicted CpG islands
Others
c)
60; 33%
120; 67%
Fully methylated CpG
Others
d)
488, 6%
7966, 94%
ATAC
Others
e)
976; 12%
7478; 88%
TF
Others
f)
131; 1%
5708; 45%
6820; 54%
TSS
Gene body
Intergenic
g)
255, 2%
12404, 98%
ATAC
Others
h)
46; 2%
979; 44%
1226; 54%
TSS
Gene Body
Intergenic
i)
62; 3%
2189; 97%
CpG island
others
j)
50; 2%
120; 5%
9; 1%
2072; 92%
Shared ATAC sites
LCL specific ATAC sites
CD34 specific ATAC sites
Shared closed chromatin
k)
571, 25%
1680, 75%
PQS
others

Supplementary Figure 3

Overlap analysis of random fragments matching in size and number the ATRX fragments under the same conditions. a. overlap with genes controlling for ATRX binding sites in LCLs. b. Overlap with CpG predicted controlling for ATRX binding sites in LCLs. c. overlap with CpG only – fully methylated vs BioCAP containing peak controlling for ATRX binding sites in LCLs. d. overlap with ATAC-seq peaks controlling for ATRX binding sites in LCLs. e. overlap with ENCODE transcription factors controlling for ATRX binding sites in LCLs. f. overlap with genes controlling for ATRX binding sites in erythroblasts. g. overlap with ATAC-seq peaks controlling for ATRX binding sites in erythroblasts. h. overlap with genes controlling for ATRX binding sites conserved in both LCLs and erythroblasts. i. overlap with CpG predicted controlling for ATRX binding sites conserved in both LCLs and erythroblasts. j. overlap with ATAC-seq peaks controlling for ATRX binding sites conserved in both LCLs and erythroblasts. k. overlap with PQS controlling for ATRX binding sites conserved in both LCLs and erythroblasts.

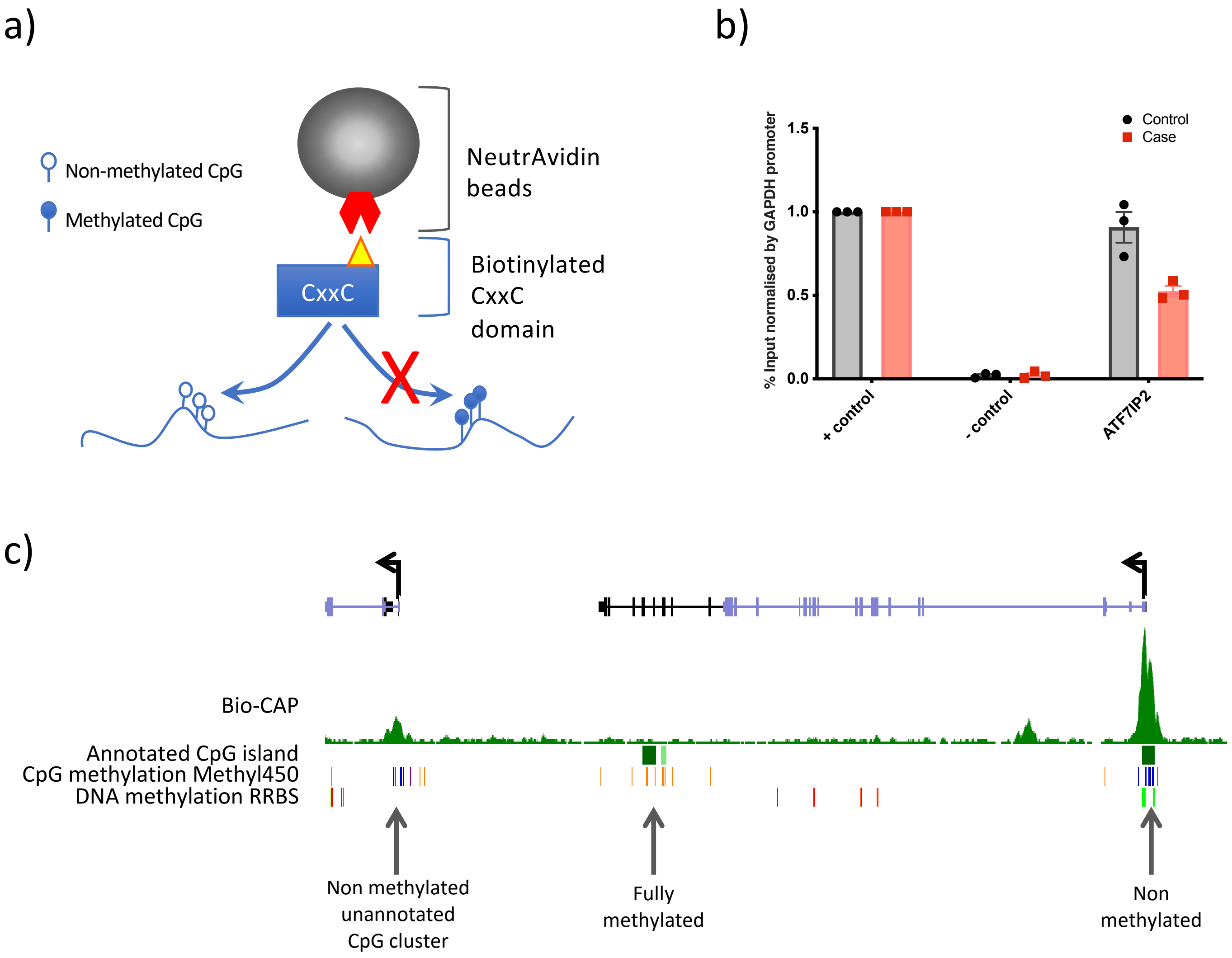

Supplementary Figure 4

Validation of the Bio-CAP-seq in LCLs. a. Schematic principle of the Bio-CAP technique (adapted from Blackledge et al., 2011). b. Validation of the Bio-CAP by qPCR (n=3 biological replicates for controls using LCLs derived from unaffected individuals and n=3 biological replicates for cases using LCLs derived from patients). + control targets an unmethylated CpG island at the *GAPDH* promoter and - control targets a region not annotated as an unmethylated CpG island. All data points are shown (bar chart represents the mean and SEM). Source data are provided as a Source Data file. c. Representative image illustrating the ability of Bio-CAP-seq to detect unmethylated CpG clusters. Bio-CAP is an average of three biological replicates using LCLs derived from three independent unaffected controls. CpG methylation Methyl450; associated colour code: Orange: methylated, Purple: partially methylated, Blue: Unmethylated. DNA methylation RRBS (Meissner et al., 2008); associated colour-code: Red: 100% methylated, Yellow: 50% methylated and Green: 0% methylated.

a)

Sites	Term	Count	pValue
active promoter	transcription, DNA-templated	512	5.88E-74
	regulation of transcription, DNA-templated	435	9.33E-76
	positive regulation of transcription from RNA polymerase II promoter	167	4.58E-06
	negative regulation of transcription from RNA polymerase II promoter	159	3.82E-14
	apoptotic process	102	4.70E-05
	negative regulation of transcription, DNA-templated	98	1.61E-06
	positive regulation of transcription, DNA-templated	89	5.81E-04
	transcription from RNA polymerase II promoter	88	7.98E-04
poised Promoter	positive regulation of GTPase activity	7	1.30E-02
	regulation of small GTPase mediated signal transduction	4	1.11E-02
active enhancers	signal transduction	131	3.01E-11
	positive regulation of transcription from RNA polymerase II promoter	85	1.97E-03
	positive regulation of GTPase activity	74	2.49E-09
	positive regulation of transcription, DNA-templated	57	3.77E-05
	intracellular signal transduction	55	9.34E-08
	apoptotic process	51	9.03E-03
	protein phosphorylation	48	5.31E-04
	negative regulation of transcription, DNA-templated	42	4.44E-02
enhancer-CTCF binding site	regulation of transcription, DNA-templated	8	1.48E-02
	transcription from RNA polymerase II promoter	5	1.26E-02
	lysosome organization	3	1.87E-03
CTCF site	homophilic cell adhesion via plasma membrane adhesion molecules	13	2.15E-16
	signal transduction	8	9.02E-03
repressed region	transcription, DNA-templated	165	3.93E-67
	regulation of transcription, DNA-templated	156	2.19E-75
	negative regulation of transcription from RNA polymerase II promoter	30	2.02E-04
	negative regulation of transcription, DNA-templated	18	1.96E-02
background	transcription, DNA-templated	80	1.68E-05
	regulation of transcription, DNA-templated	68	3.67E-06
	signal transduction	47	1.77E-03
	positive regulation of transcription from RNA polymerase II promoter	43	6.39E-04
	positive regulation of GTPase activity	28	1.28E-03
	negative regulation of transcription from RNA polymerase II promoter	28	2.83E-02
	protein phosphorylation	27	1.09E-04

c)

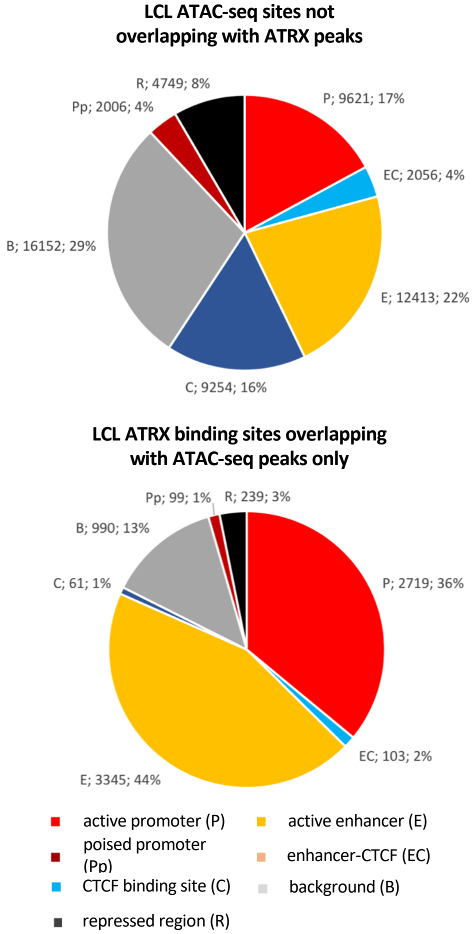

b)

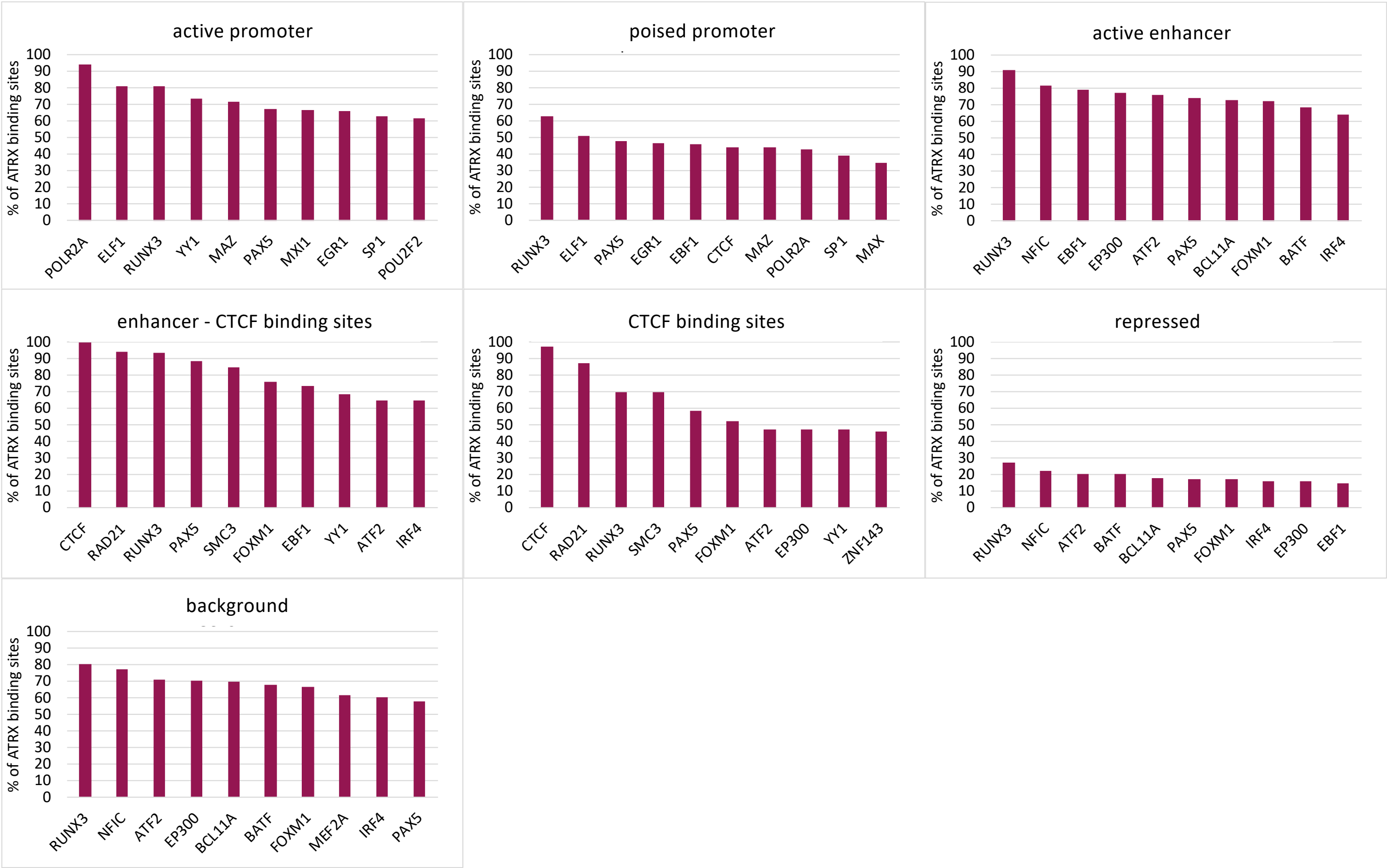

Supplementary Figure 5

Analysis of the subclasses of ATRX binding sites in LCLs based on the GenoSTAN annotation. a. GO analysis of ATRX binding sites based on GenoSTAN subclasses in LCLs. b. Enrichment analysis for the transcription factors based on their percentage overlap at ATRX binding sites subdivided in GenoSTAN subclasses in LCLs (showing only the top 10 most enriched) p-values based on EASE Score DAVID 6.8 (Da Wei Huang et al., 2008). GenoSTAN analysis in LCLs of: (top) all ATAC-seq sites identified in LCLs which were not overlapping with ATRX binding sites, (bottom) all the ATRX binding sites identified in LCLs containing at least one open chromatin region - active promoter (P), poised promoter (Pp), active enhancer (E), enhancer-CTCF binding site (EC), CTCF binding site (C), repressed region (R) and background (B).

a)

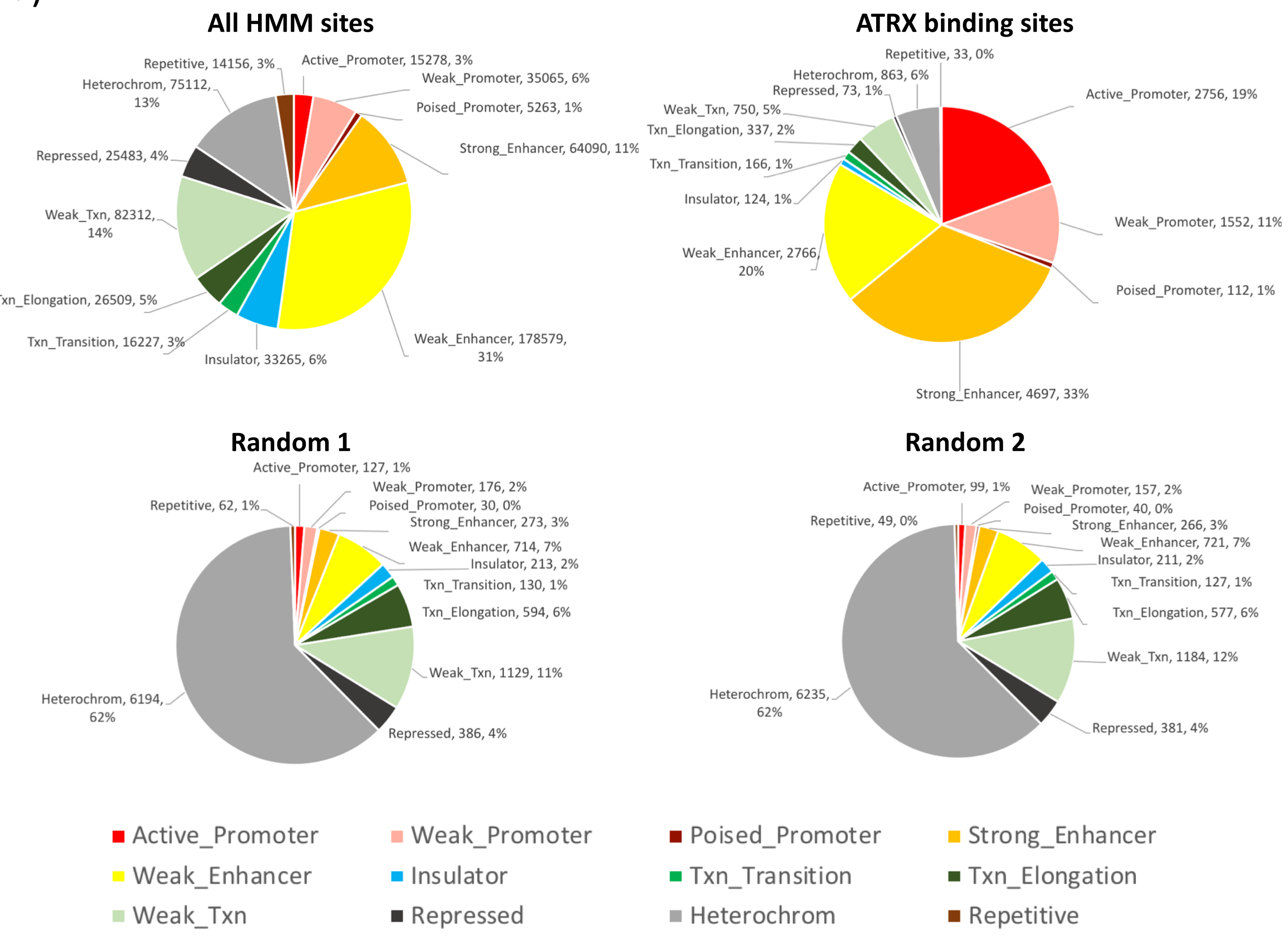

b)

Rank	Motif	Name	P-value	% of Targets
1	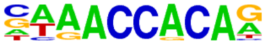	RUNX (Runt)	1.00E-39	15.71%
2	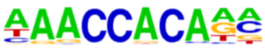	RUNX1 (Runt)	1.00E-31	19.56%
3	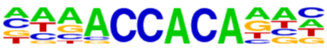	RUNX2 (Runt)	1.00E-27	16.25%

c)

Rank	Motif	Name	p-value	% of Targets
1	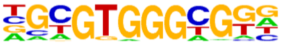	Egr2(Zf)	1.00E-11	5.54%
2	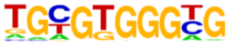	Egr1(Zf)	1.00E-05	11.19%
3	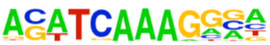	Tcf4	1.00E-05	7.22%

d)

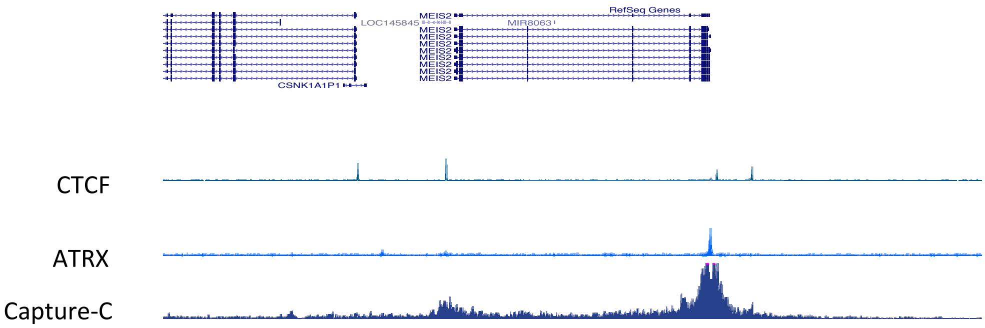

Supplementary Figure 6

Analysis of TFBS and motifs at ATRX binding sites. a. HMM analysis of: (Top left) all the HMM sites from ENCODE data set for LCLs, (Top right) the ATRX binding sites within this data set, (Bottom) two randomly selected sets of regions. b. Motif analysis of known motifs contained at the subset of ATRX binding sites, which contained at least one open chromatin region and using the ATAC-seq peaks not associated with ATRX as background. p-values HOMER findMotifsGenome.pl (Heinz et al., 2010). c. Motif analysis of known motifs contained at the subset of ATRX binding sites which did not contain open chromatin regions, and using random sequences from the human genome as background p-values HOMER findMotifsGenome.pl (Heinz et al., 2010). d. Representative image of a Capture-C experiment using the ATRX enriched promoter of *MEIS2* as the view point showing that ATRX was not enriched at the promoter interacting region downstream *MEIS2*.

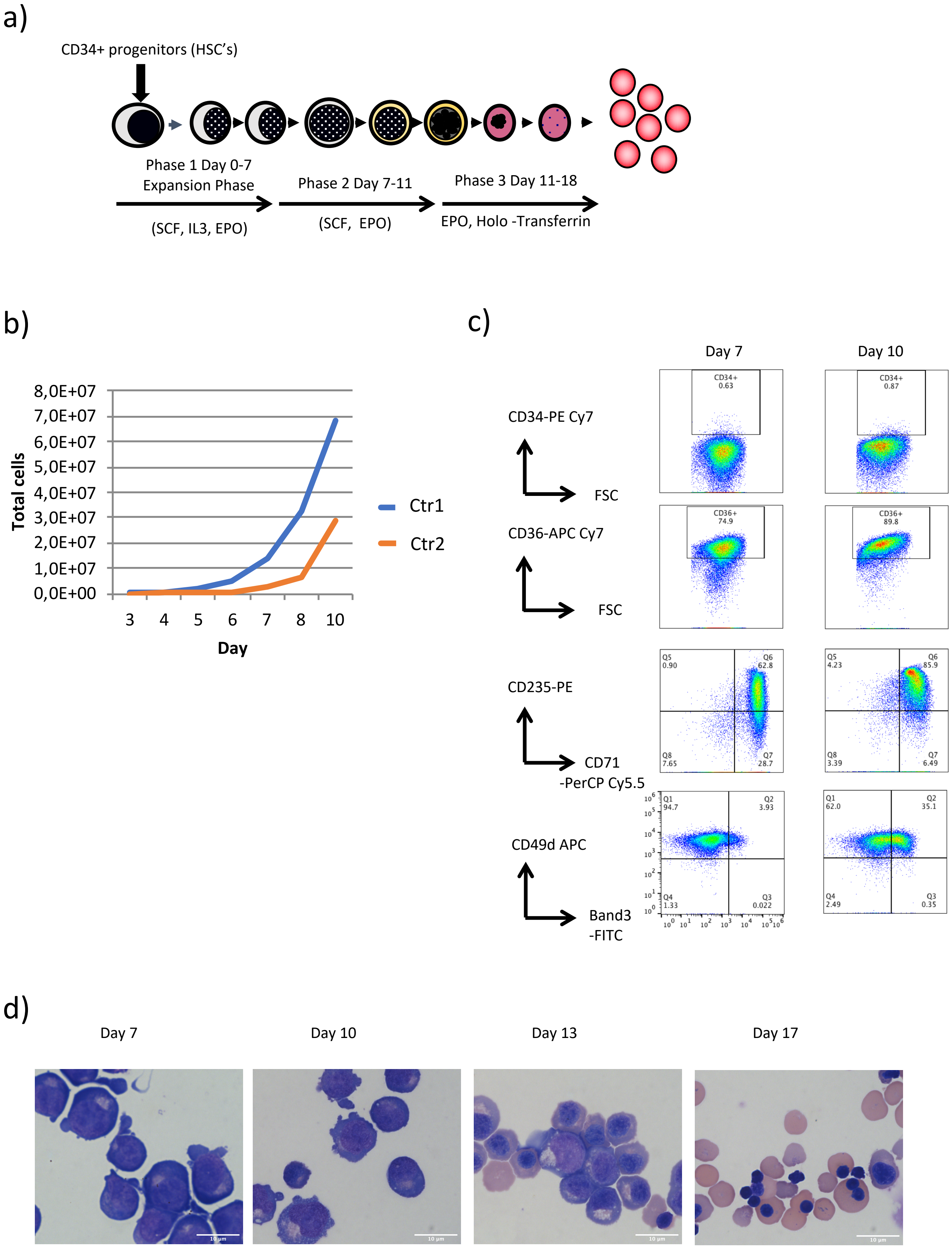

a)
CD34+ progenitors (HSC's)
Phase 1 Day 0-7
Expansion Phase
(SCF, IL3, EPO)
Phase 2 Day 7-11
(SCF, EPO)
Phase 3 Day 11-18
EPO, Holo -Transferrin
b)
Total cells
8,0E+07
7,0E+07
6,0E+07
5,0E+07
4,0E+07
3,0E+07
2,0E+07
1,0E+07
0,0E+00
3
4
5
6
7
8
10
Day
Ctr1
Ctr2
c)
Day 7
Day 10
CD34-PE Cy7
FSC
CD34+ 0.63
CD34+ 0.87
CD36-APC Cy7
FSC
CD36+ 74.9
CD36+ 89.8
CD235-PE
CD71 -PerCP Cy5.5
Q5 0.90
Q6 62.8
Q8 7.65
Q7 28.7
Q5 4.23
Q6 85.9
Q8 3.39
Q7 6.49
CD49d APC
Band3 -FITC
Q1 94.7
Q2 3.93
Q4 1.33
Q3 0.022
Q1 62.0
Q2 35.1
Q4 2.49
Q3 0.35
d)
Day 7
Day 10
Day 13
Day 17

Supplementary Figure 7

Differentiation of CD34+ HSPCs. a. Overview of the protocol for the differentiation of the CD34+ HSPCs. b. Proliferation curve of differentiating CD34+ HSPCs from two normal donors, Ctr1 and Ctr2. Source data are provided as a Source Data file. c. FACS analysis of differentiating CD34+ HSPCs using varous markers expressed in terminal erythroid differentiation d. Cytospins of differentiating CD34+ HSPCs (scale bar = 10 µm). Ctr 1 has been differentiated twice (n=2) and Ctr 2 has been differentiated three times (n=3).

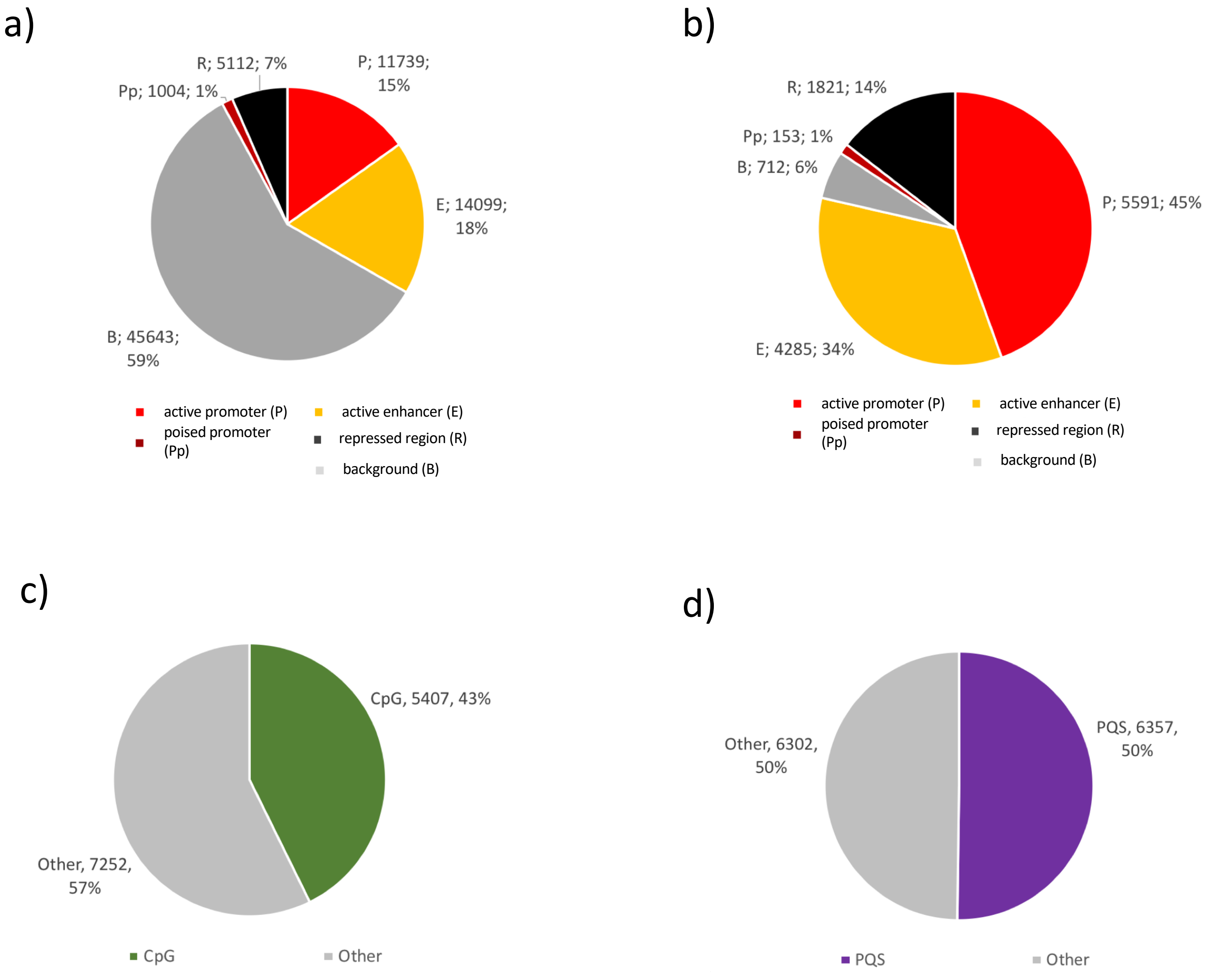

Supplementary Figure 8

ATRX enrichment in cultured erythroblasts. (a and b) Identification of regulatory elements at open chromatin sites in normal donor day 10 CD34+ HSPCs using GenoSTan. a. Pie chart representing the distribution of the identified chromatin states at all the open chromatin sites included in the dataset: active promoter (P), poised promoter (Pp), active enhancers (E), repressed region (R) and background (B). b. Pie chart representing the distribution of the identified chromatin states at the ATRX binding sites spanning open chromatin regions only. c. Distribution of the ATRX binding sites based on their position relative to CpG islands in erythroblasts. d. Distribution of the PQS and non-PQS containing sequences at ATRX binding sites in erythroblasts.

a)

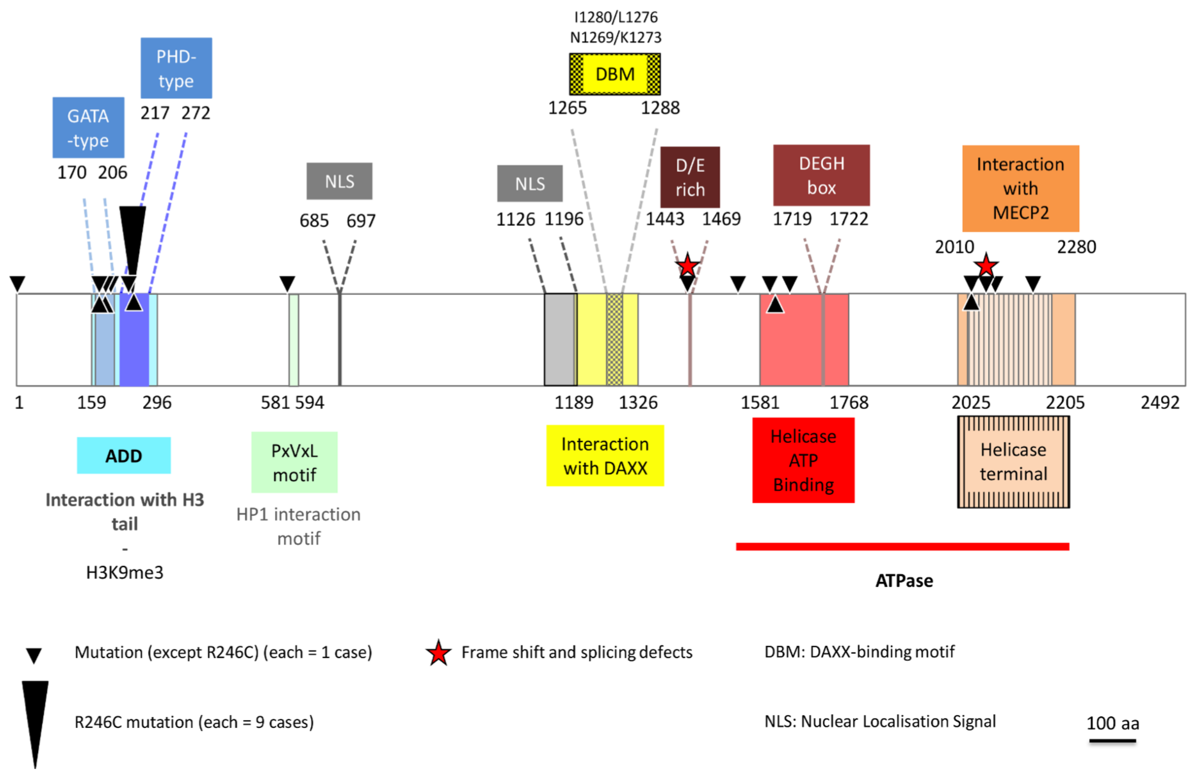

b)

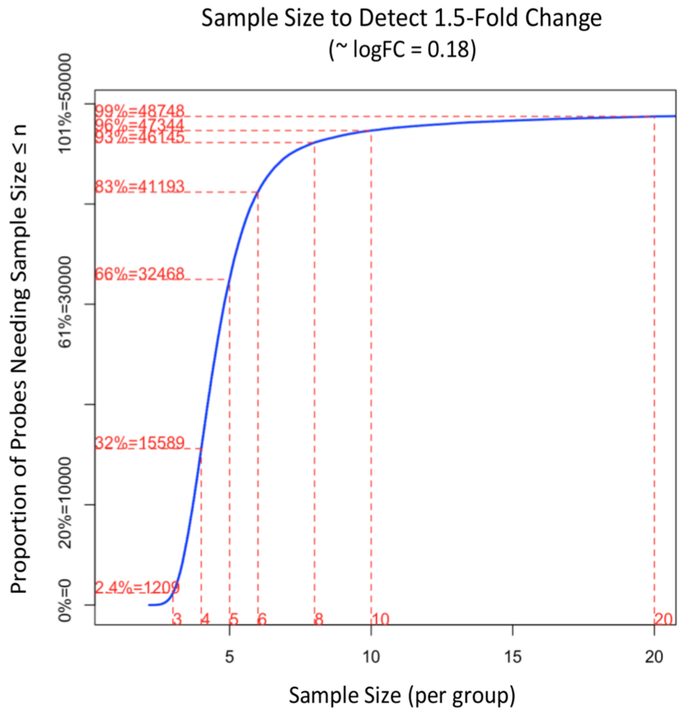

Supplementary Figure 9

Experimental design for the microarray analysis. a. Location of the ATRX mutations in selected ATR-X cases. b. Power calculation

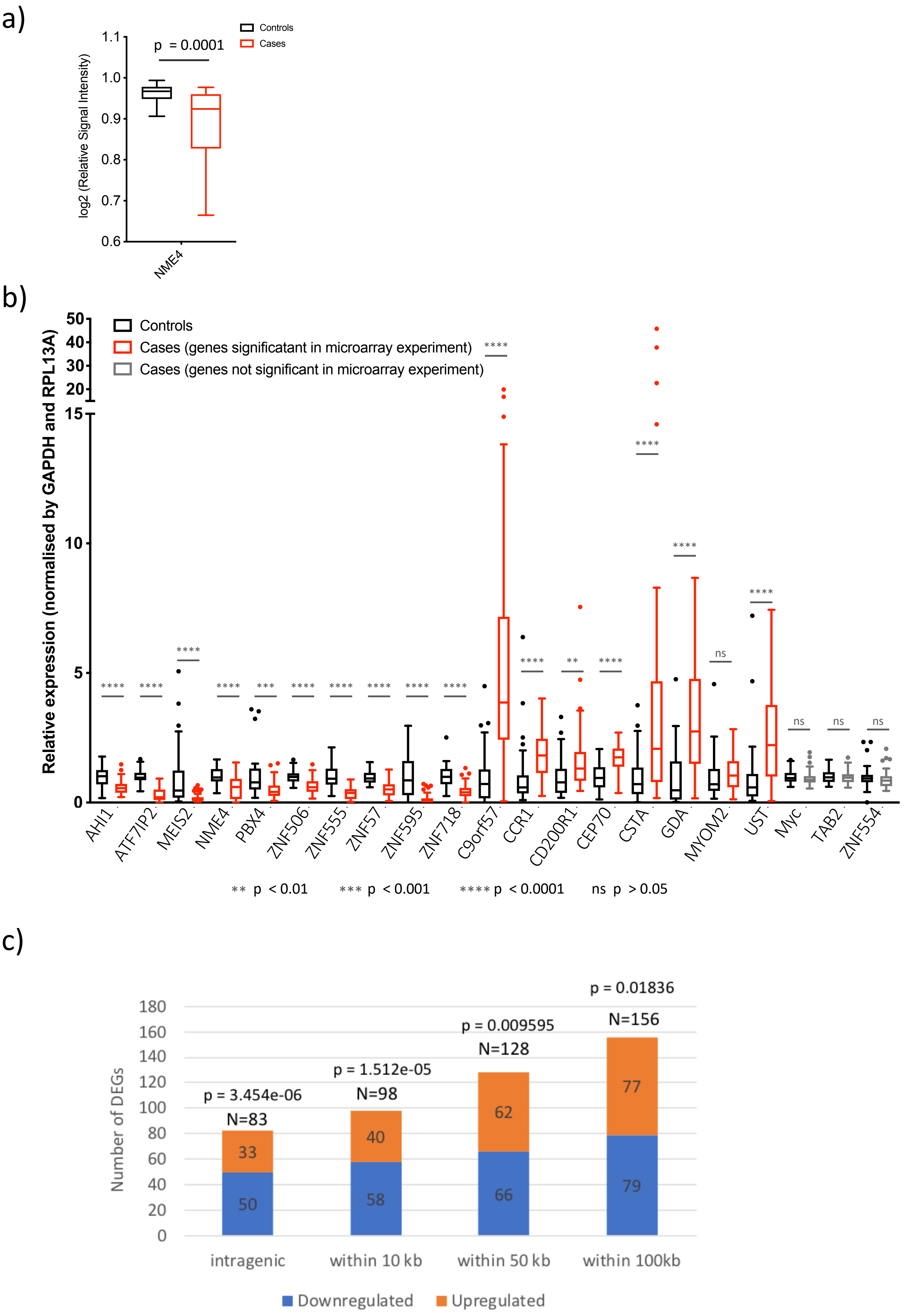

a)
log2 (Relative Signal Intensity)
1.1
1.0
0.9
0.8
0.7
0.6
p = 0.0001
Controls
Cases
NME4
b)
Relative expression (normalised by GAPDH and RPL13A)
50
40
30
20
15
10
5
0
Controls
Cases (genes significatant in microarray experiment)
Cases (genes not significant in microarray experiment)
AHI1
ATF7IP2
MEIS2
NME4
PBX4
ZNF506
ZNF555
ZNF57
ZNF595
ZNF718
C9orf57
CCR1
CD200R1
CEP70
CSTA
GDA
MYOM2
UST
Myc
TAB2
ZNF554
** p < 0.01
*** p < 0.001
**** p < 0.0001
ns p > 0.05
c)
Number of DEGs
180
160
140
120
100
80
60
40
20
0
p = 3.454e-06
N=83
33
50
p = 1.512e-05
N=98
40
58
p = 0.009595
N=128
62
66
p = 0.01836
N=156
77
79
intragenic
within 10 kb
within 50 kb
within 100kb
Downregulated
Upregulated

Supplementary Figure 10

ATRX mutations are associated with change in gene expression. a. Relative expression of *NME4* in microarrays comparing ATR-X cases and normal controls. Tukey based box plot showing the 25th and 75th percentiles (lower and upper bounds of the box, respectively), the median (centre line), the minimum value lower than the 25th percentile minus 1.5*IQR (lower whisker) and the maximum value greater than the 75th percentile plus 1.5*IQR (upper whisker), any values beyond the whiskers boundaries are represented as dots, p-values based on Mann-Whitney test, two-tailed (n=20 biological replicates for controls using LCLs derived from unaffected individuals and n=28 biological replicates for cases using LCLs derived from patients). b. Validation of the microarray data by high-throughput qPCR. Tukey based box plot showing the 25th and 75th percentiles (lower and upper bounds of the box), the median (centre line), the minimum value greater than the 25th percentile minus 1.5*IQR (lower whisker) and the maximum value greater than the 75th percentile plus 1.5*IQR (upper whisker), any values beyond the whiskers boundaries are represented as dots, p-values based on Mann-Whitney test, two-tailed (n=34 for controls (two independent replicates of RNA extraction experiments using 17 controls) and n=46 for cases (two independent replicates of RNA extraction experiments using 23 controls)). p-values <0.0001 for *AHI1*, *ATF7IP2*, *MEIS2*, *NME4*, *ZNF506*, *ZNF555*, *ZNF57*, *ZNF595*, *ZNF718*, *C9orf57*, *CCR1*, *CEP70*, *CSTA*, *GDA* and *UST* and p-values for *PBX4*=0.002, *CD200R1*=0.0015, *MYOM2*=0.1258, *Myc*=0.2883, *ZNF554*=0.1605 and *TAB2*=0.5451. c. Distribution of the differentially expressed genes (DEGs) relative to the position of the closest ATRX binding sites and the change in gene expression in LCLs derived from ATR-X cases compared to controls (intragenic: p-value = 3.454e-06, odds ratio = 1.95, intragenic and 10kb region: p-value = 1.512e-05, odds ratio = 1.81, intragenic and 50kb region: p-value = 0.009595, odds ratio = 1.41, intragenic and 100kb region: p-value = 0.01836, odds ratio = 1.39, Fisher's Exact Test, two-sided) .

a)

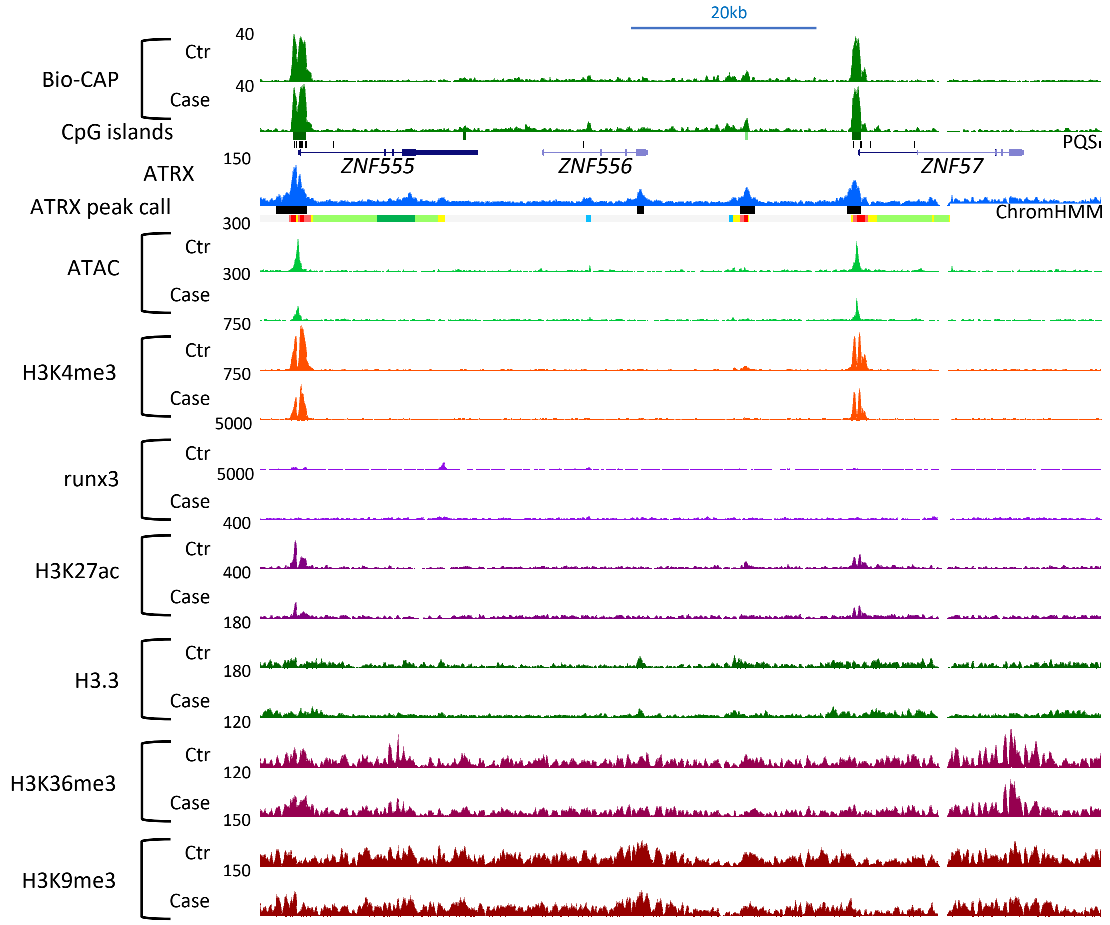

b)

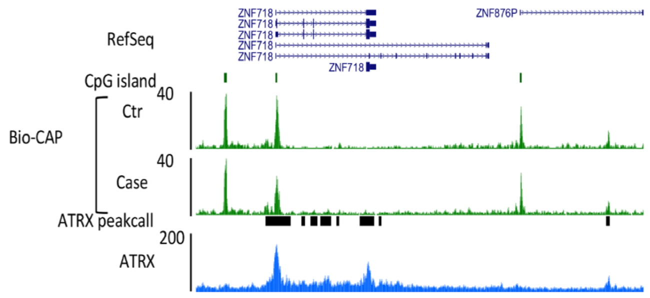

Supplementary Figure 11

Pathogenic ATRX mutations associated with changes in the chromatin environment of a. *ZNF555* and *ZNF57* and, b. *ZNF718*. The signals represent an average of the independent replicates (n=6 for ATRX, n=3 for H3K4me1, H3K4me3 and H3.3 ChIP-seq and Bio-CAP-seq, n =2 for H3K27ac and runx3 ChIP-seq and n=4 for ATAC-seq).

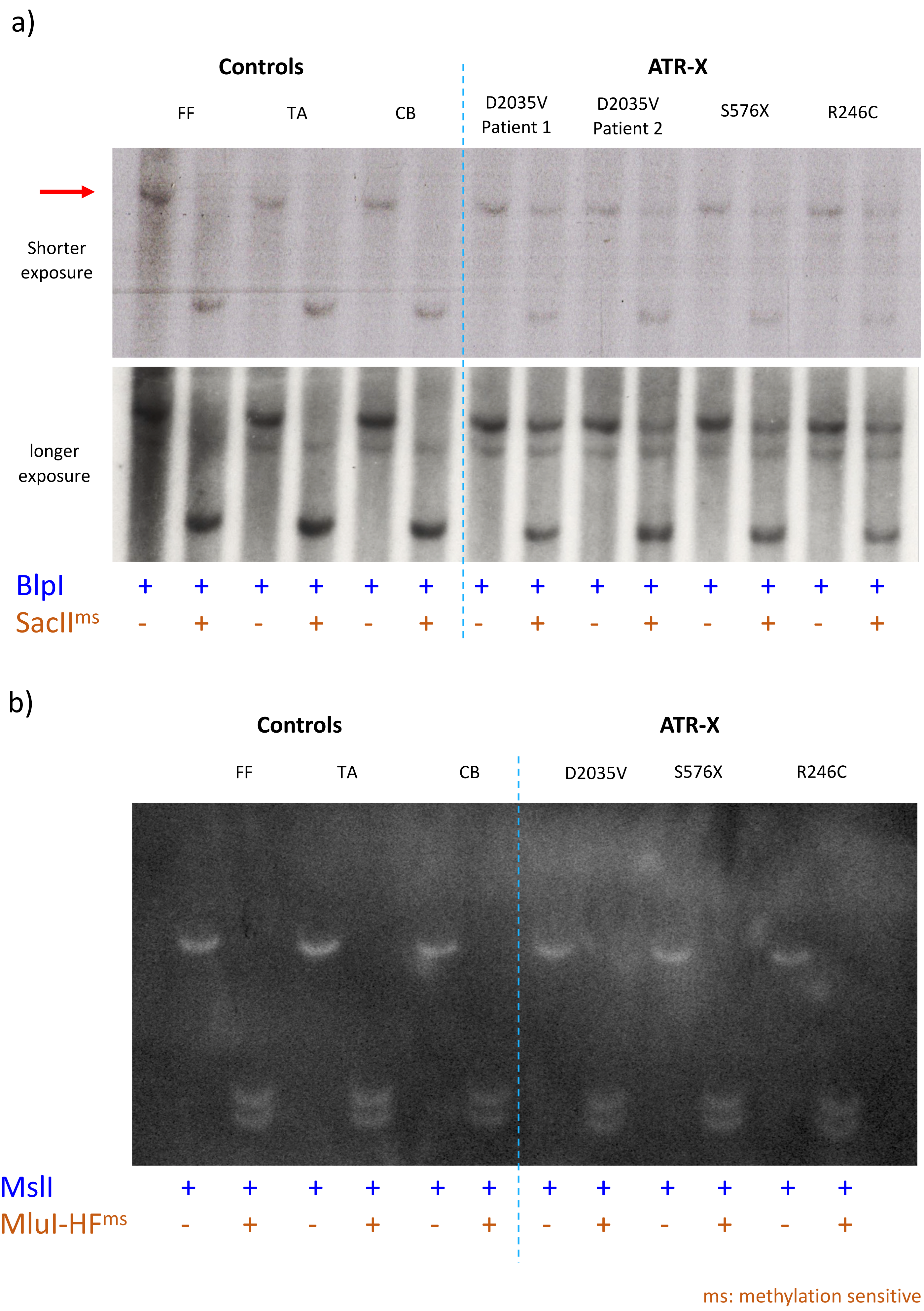
a)
Controls
ATR-X
FF
TA
CB
D2035V Patient 1
D2035V Patient 2
S576X
R246C
Shorter exposure
longer exposure
BlpI
+ + + + + + + + + + + + + +
SacII^ms
- + - + - + - + - + - + - +
b)
Controls
ATR-X
FF
TA
CB
D2035V
S576X
R246C
MslI
+ + + + + + + + + + + +
MluI-HF^ms
- + - + - + - + - + - +
ms: methylation sensitive

Supplementary Figure 12

Southern blots assessing the methylation status at the promoter region of a. *ATF7IP2* (n=3 for controls and n=4 for cases) and b. *ZNF555* (n=3 for controls and n=3 for cases). For *ATF71P2*, each sample is subject to a limit digest with BlpI. Subsequent digestion with the methylation sensitive enzyme SacII indicates the methylation status of the locus. Unmethylated sites are digested to a smaller fragment whereas methylated loci are not further digested by SacII and a limit digest fragment persists, indicated by the red arrow seen in the ATR-X cases. At *ZNF555* the limit digest used is MslI and the methylation sensitive enzyme is MluI. Source data are provided as a Source Data file.

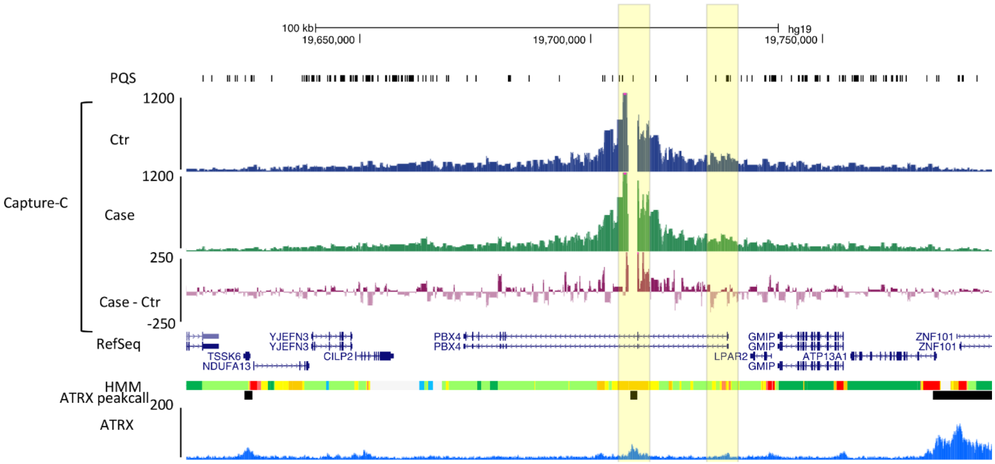

Supplementary Figure 13

Chromatin interaction at the *PBX4* locus. a. Chromatin interaction observed by Capture-C showing the interaction between the *PBX4* promoter and a putative enhancer (marked by yellow boxes) bound by ATRX in LCLs. The signals represent an average of the independent replicates (n=6 for ATRX, n=3 for Capture-C).

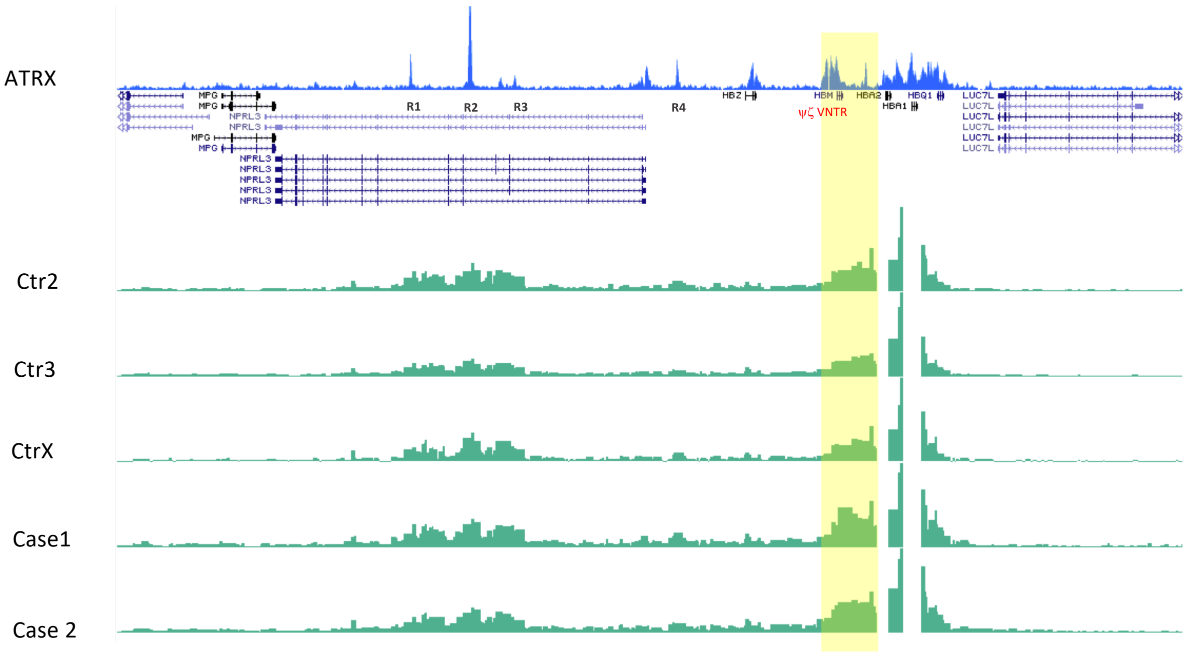

Supplementary Figure 14

Capture-C analysis at the alpha-globin cluster (marked by a yellow box) using *HBA1/2* as viewpoint comparing interactions between the promoters and enhancers for ATR-X cases (case1 and case2) and controls (Ctr2, Ctr3 and CtrX). n=1 for Capture-C and n=4 for ATRX ChIP-seq (average signal displayed).

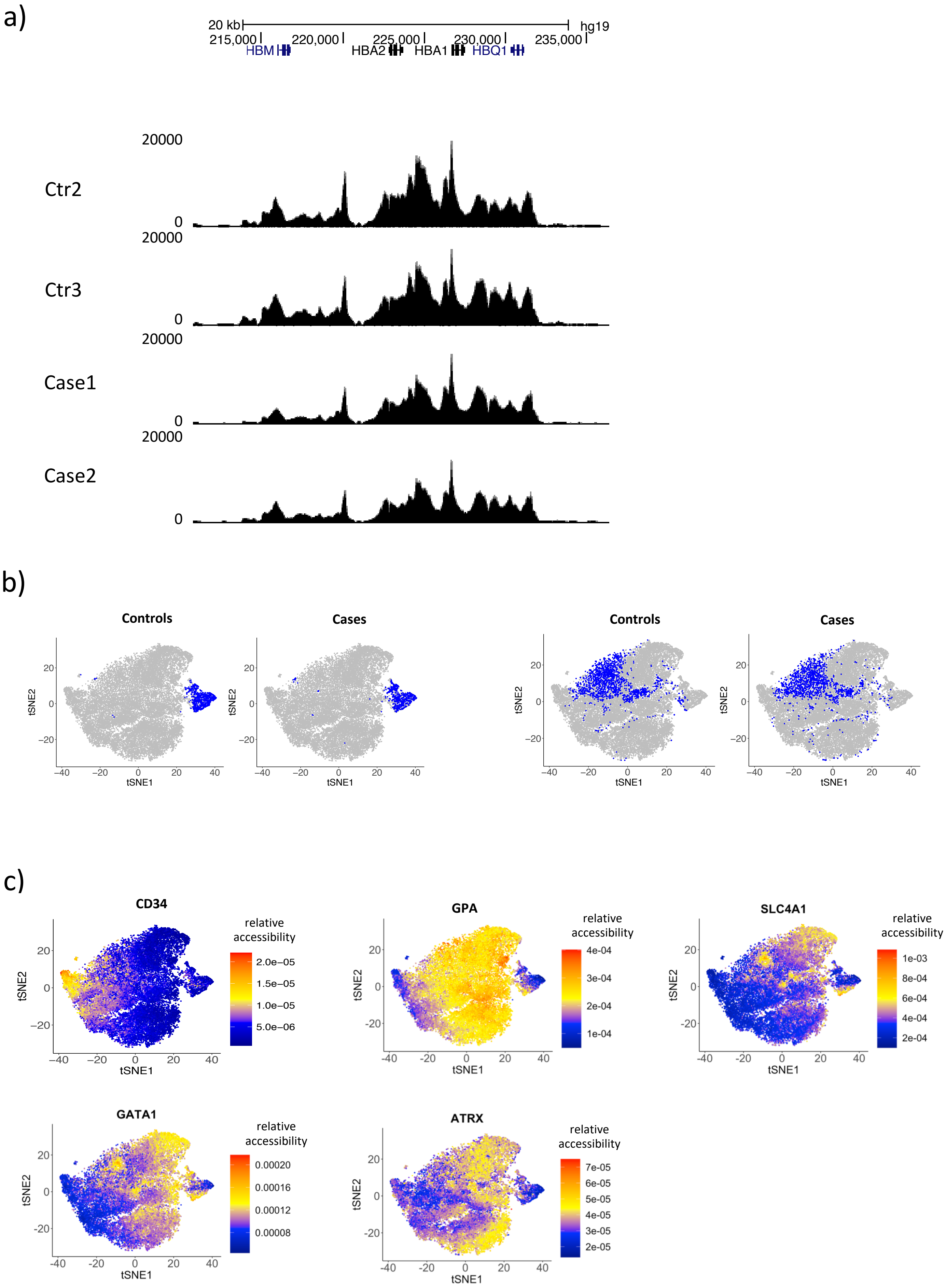
a)
20 kb
hg19
215,000
220,000
225,000
230,000
235,000
HBM
HBA2
HBA1
HBQ1
Ctr2
Ctr3
Case1
Case2
20000
0
b)
Controls
Cases
Controls
Cases
tSNE1
tSNE2
c)
CD34
GPA
SLC4A1
GATA1
ATRX
relative accessibility
2.0e−05
1.5e−05
1.0e−05
5.0e−06
4e-04
3e-04
2e-04
1e-04
1e-03
8e-04
6e-04
4e-04
2e-04
0.00020
0.00016
0.00012
0.00008
7e-05
6e-05
5e-05
4e-05
3e-05
2e-05
tSNE1
tSNE2

Supplementary Figure 15

scATAC-seq analysis of erythroblasts from ATR-X cases. a. scATAC-seq plotted as bulk across the HBM and HBA loci showing a reduced signal in ATR-X cases (Case1 and Case2) compare to controls (Ctr2 and Ctr3) (n=1). b. t-SNE analysis of the scATAC-seq highlighting the cells in control and case in clusters containing the regions pointed by arrows in figure 5e-g. c. t-SNE analysis of scATAC-seq data showing each individual cell and highlighting in the chromatin accessibility score for the genes encoding the markers of erythroid differentiation CD34 (early), GPA (intermediate) and SLC4A1 (late) and the transcription factors GATA1 and ATRX.

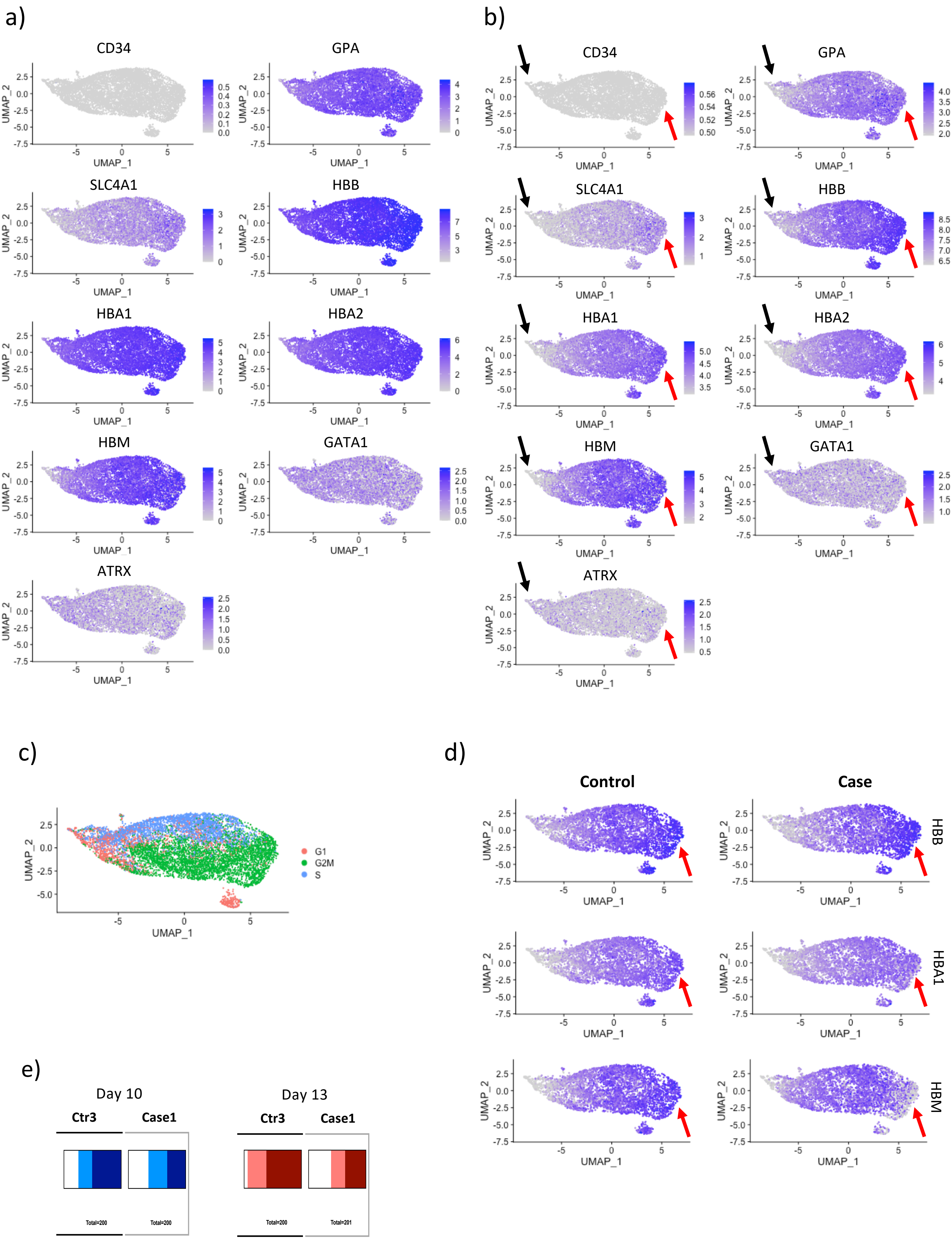

a)
CD34
GPA
SLC4A1
HBB
HBA1
HBA2
HBM
GATA1
ATRX
UMAP_1
UMAP_2
b)
CD34
GPA
SLC4A1
HBB
HBA1
HBA2
HBM
GATA1
ATRX
c)
G1
G2M
S
d)
Control
Case
HBB
HBA1
HBM
e)
Day 10
Day 13
Ctr3
Case1
Ctr3
Case1
Total=200
Total=200
Total=200
Total=201

Supplementary Figure 16

Single cell analysis of erythroblasts from ATR-X case and unaffected donor. a and b. UMAP analysis of scRNA-seq data (control and case) showing each individual cell and highlighting the relative expression signals for the genes encoding the markers of erythroid differentiation CD34 (early), GPA (intermediate) and SLC4A1 (late), HBB, HBA and HBM and the transcription factors GATA1 and ATRX. In a. all signals and in b. signals above quantile 5 (black arrow pointing the left side of the main population where cells tend to be slightly less differentiated by contrast with red arrow pointing the right side of the main population where cells tend to be slightly more differentiated). c. UMAP analysis of scRNA-seq data highlighting the cell cycle phase for each cell. d. UMAP analysis of scRNA-seq data comparing control and case and highlighting the relative expression signals (above quantile 5) for *HBB*, *HBA* and *HBM* (red arrow pointing the right side of the main population where cells have a high expression signal for *HBB* but a reduce signal for *HBA* and *HBM* in case compare to control). e. Analysis of alpha-globin expression by RNA FISH at days 10 and 13 showing in white the cells with no allelic activity of *HBA* detected, in light blue (control) and light red (ATR-X case) the cells with one allelic signal of HBA detected and in dark blue (control) and dark red (ATR-X case) cells with two allelic signals detected.

a)

	Protein	Channel	Supplier	Catalogue Number	Volume (ul) used in 100ul
**CD235a**	Glycophorin A	PE	BD Bioscience	555570	1
**CD71**	Transferrin	PerCP Cy5.5	Biolegend	334114	1
**CD49D**	α-Integrin	APC	BD Bioscience	561892	2
**CD34**	CD34	PE/Cy7	Biolegend	343616	1
**CD233**	Band3	FITC	IBGRL	9439FI	1
**CD36**	Platelet glycoprotein	APC/Cy7	Biolegend	336213	1
**Hoechst 33258**	viability dye	violet	Invitrogen	H3569	1 in 10000 dilution

b)

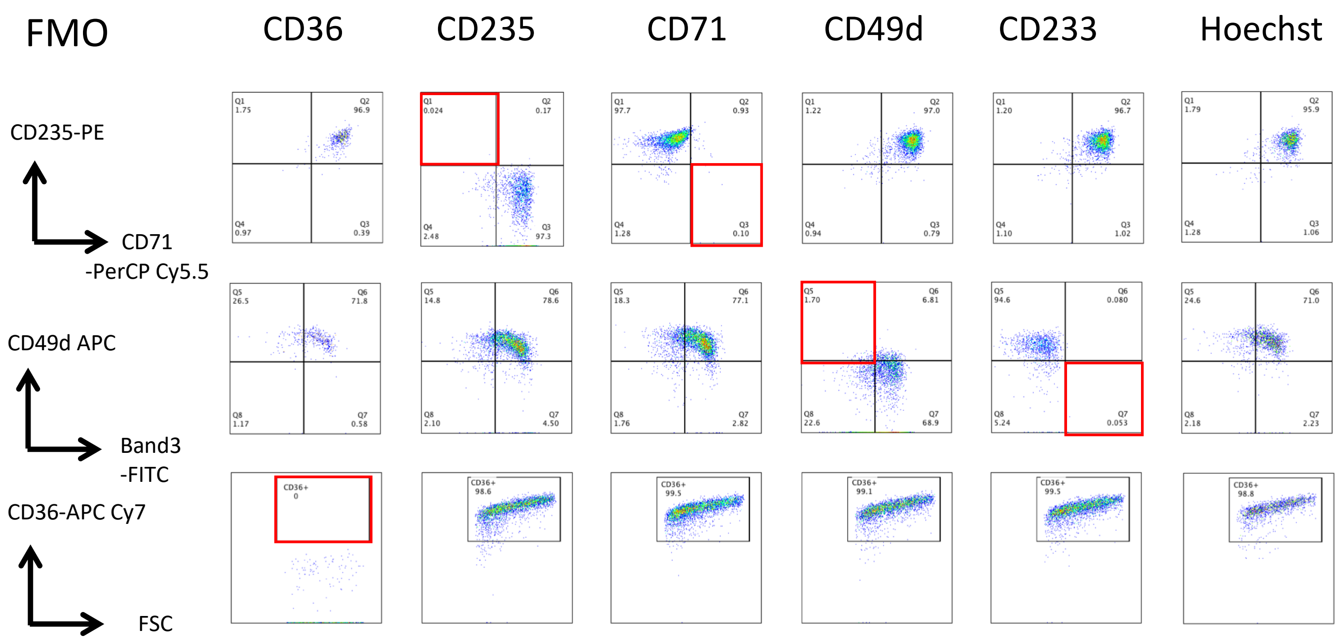

Supplementary Figure 17

Information regarding flow cytometry. a. Antibodies used for FACS experiments. b. Fluorescence minus one (FMO) controls for Flow cytometry analysis. Gates were set for each population on FMO. Red boxes indicate the positive population for each marker.

Gene List	Term	Count	p-value	Genes
DEGs p < 0.05 |logFC| > 0.5	regulation of transcription, DNA-templated	15	0.020	ZNF595, ZNF555, L3MBTL4, ZNF506, ZNF57, ZNF626, ATF7IP2, RPS6KA5, ZNF30, HOXA1, ZNF718, STAT4, MEIS2, LBH, TCFL5
	lipid transport	4	0.007	NME4, APOL2, APOL1, ABCA6
	central nervous system development	4	0.024	NDN, AHI1, VCAN, PDX1
DEGs p < 0.05 |logFC| > 0.5 Intragenic ATRX binding site	regulation of transcription, DNA-templated	11	4.48E-05	ZNF555, ZNF30, ZNF595, ZNF718, L3MBTL4, LBH, MEIS2, ZNF506, ZNF57, ZNF626, ATF7IP2
	transcription, DNA-templated	11	4.07E-04	ZNF555, ZNF30, ZNF595, ZNF718, L3MBTL4, LBH, ZNF506, ZNF57, ZNF626, ATF7IP2, ZBTB38
DEGs p < 0.05 |logFC| > 0.5 ATRX binding site within 10kb	regulation of transcription, DNA-templated	12	1.58E-04	ZNF595, LBH, ZNF718, ZNF506, ZNF626, ZNF30, ATF7IP2, STAT4, ZNF57, ZNF555, MEIS2, L3MBTL4
	transcription, DNA-templated	12	0.002	ZNF595, LBH, ZNF718, ZNF506, ZBTB38, ZNF626, ZNF30, ATF7IP2, STAT4, ZNF57, ZNF555, L3MBTL4
	negative regulation of gene expression	3	0.033	CCR1, CD28, AIF1
DEGs p < 0.05 |logFC| > 0.5 ATRX binding site within 50kb	regulation of transcription, DNA-templated	13	1.32E-04	ZNF595, ATF7IP2, ZNF57, MEIS2, LBH, ZNF718, ZNF506, ZNF626, ZNF30, STAT4, ZNF555, L3MBTL4, TCFL5
	transcription, DNA-templated	13	0.001	ZNF595, ZBTB38, ATF7IP2, ZNF57, LBH, ZNF718, ZNF506, ZNF626, ZNF30, STAT4, TXNIP, ZNF555, L3MBTL4
	negative regulation of gene expression	3	0.042	CCR1, CD28, AIF1
DEGs p < 0.05 |logFC| > 0.5 ATRX binding site within 100kb	regulation of transcription, DNA-templated	13	0.001	ZNF555, ZNF30, ZNF595, ZNF718, L3MBTL4, STAT4, MEIS2, LBH, TCFL5, ZNF506, ZNF57, ZNF626, ATF7IP2
	transcription, DNA-templated	13	0.009	ZNF555, TXNIP, ZNF30, ZNF595, ZNF718, L3MBTL4, STAT4, LBH, ZNF506, ZNF57, ZNF626, ATF7IP2, ZBTB38

Supplementary Table 1

Gene ontology analysis based on the microarray data analysis from LCLs, ATR-X cases vs unaffected donors showing the top enriched pathways based on the count with a p-value < 0.05 for the DEGs with a |logFC| > 0.5 (p-values based on EASE Score DAVID 6.8 (Da Wei Huang et al., 2008).

Gene Symbol	Assay ID
ACTB	Hs01060665_g1
AHI1	Hs01053982_m1
ATF7IP2	Hs01101353_m1
C9orf57	Hs01370396_m1
CCR1	Hs00928897_s1
CD200R1	Hs00793597_m1
CEP70	Hs00225806_m1
CSTA	Hs00193257_m1
GAPDH	Hs03929097_g1
GDA	Hs00188737_m1
MEIS2	Hs00542636_m1
MYC	Hs00153408_m1
MYOM2	Hs01556320_m1
NME4	Hs00359037_m1
PBX4	Hs00257935_m1
RPL13A	Hs04194366_g1
TAB2	Hs00248373_m1
UST	Hs01053964_m1
ZNF506	Hs00869072_g1
ZNF554	Hs01014440_m1
ZNF555	Hs00328296_s1
ZNF57	Hs00380549_m1
ZNF595	Hs00745818_s1
ZNF718	Hs00799585_sH

Supplementary Table 2

TaqMan® Assays from ThermoFisher Scientific

Name	Forward	Reverse
ATF7IP2	GCTGCCATTGGTCCATAATC	CCTTGGCTGCTTGTGATTTC
DIST	GAGATGCTGGAGTCAGGACCAT	AGGAGTCAGGAGCAGCAGTCA
G4 negative/CpG island negative control	GCAAAAGCGTTTGGGTATTC	TGTCCTTTCAAAAGGCCTACC
GAPDH promoter	TATAAATTGAGCCCGCAGCCT	AAAGAAGATGCGGCTGACTGT
rDNA	CGGGCGTGGAATGCGAGTG	GGGGTCTGATGAGCGTCGGC
UST	CGCCCACTTTCTTTTCTCAG	AACAGAGCAAGGCAACACAG
ZNF506	AGCTGGGCAATGAGAACTTG	AGAGTGCCAGTTCGACATCC
ZNF555	GGAACTGCCCAATTTACAGC	TGATTAGACAGAGCTCGGGAAG
ZNF57	GTCCGGAAAACTGTCCAATC	TTGGACACTACTGCAAACCTG
ZNF718	AGGCCCATCAAAACATGG	GATTTGCTTCGCTTCCTCAC

Supplementary Table 3

List of primers used for qPCR

cell type	experiment	total number of replicates	sample source - cells derived from:	sample 1	sample 2	sample 3	sample 4	sample 5	sample 6	Note
**LCLs**	ATRX ChIP-seq control	6	3 different unaffected donors using 2 different antibodies (6 in total)	FF_Ctr_LCL_ATRX_H300	TA_Ctr_LCL_ATRX_H300	CB_Ctr_LCL_ATRX_H300	FF_Ctr_LCL_ATRX_Abcam	TA_Ctr_LCL_ATRX_Abcam	CB_Ctr_LCL_ATRX_Abcam	
**LCLs**	input control (two steps cross linking)	6	3 different unaffected donors using 2 different antibodies (6 in total)	FF_Ctr_LCL_Input_DC_H300	TA_Ctr_LCL_Input_DC_H300	CB_Ctr_LCL_Input_DC_H300	FF_Ctr_LCL_Input_DC_Abcam	TA_Ctr_LCL_Input_DC_Abcam	CB_Ctr_LCL_Input_DC_Abcam	
**LCLs**	runx3 ChIP-seq control	2	the same unaffected donor (father of the ATR-X case 1)	Ctr1_rep1_LCL_Runx3	Ctr1_rep2_LCL_Runx3					
**LCLs**	runx3 ChIP-seq case	2	the same patient	Case1_rep1_LCL_Runx3	Case1_rep2_LCL_Runx3					
**LCLs**	H3K9me3 ChIP-seq control	3	3 different unaffected donors	Ctr1_LCL_H3K9me3	Ctr2_LCL_H3K9me3	Ctr3_LCL_H3K9me3				
**LCLs**	H3K9me3 ChIP-seq case	3	3 different patients	Case1_LCL_H3K9me3	Case2_LCL_H3K9me3	Case3_LCL_H3K9me3				
**LCLs**	input control (one step cross linking)	3	3 different unaffected donors	Ctr1_LCL_SC_input	Ctr2_LCL_SC_input	Ctr3_LCL_SC_input				
**LCLs**	input case (one step cross linking)	3	3 different patients	Case1_LCL_SC_input	Case2_LCL_SC_input	Case3_LCL_SC_input				
**LCLs**	H3K4me3 ChIP-seq control	4	3 different unaffected donors (see note)	Ctr1_LCL_H3K4me3	Ctr2_LCL_H3K4me3	Ctr3_LCL_H3K4me3	Ctr1_rep2_LCL_H3K4me3			Ctr1_rep2_LCL_H3K4me3 was performed independently to assess the replication of Ctr1_LCL_H3K4me3 experiment but it is not included in the average representation of the control
**LCLs**	H3K4me3 ChIP-seq case	4	3 different patients (see note)	Case1_LCL_H3K4me3	Case2_LCL_H3K4me3	Case3_LCL_H3K4me3	Case1_rep2_LCL_H3K4me3			Case1_rep2_LCL_H3K4me3 was performed independently to assess the replication of Case1_LCL_H3K4me3 experiment but it is not included in the average representation of the case
**LCLs**	H3K4me1 ChIP-seq control	3	3 different unaffected donors	Ctr1_LCL_H3K4me1	Ctr2_LCL_H3K4me1	Ctr3_LCL_H3K4me1				
**LCLs**	H3K4me1 ChIP-seq case	3	3 different patients	Case1_LCL_H3K4me1	Case2_LCL_H3K4me1	Case3_LCL_H3K4me1				
**LCLs**	H3K36me3 ChIP-seq control	3	3 different unaffected donors	Ctr1_LCL_H3K36me3	Ctr2_LCL_H3K36me3	Ctr3_LCL_H3K36me3				
**LCLs**	H3K36me3 ChIP-seq case	3	3 different patients	Case1_LCL_H3K36me3	Case2_LCL_H3K36me3	Case3_LCL_H3K36me3				
**LCLs**	H3.3 ChIP-seq control	3	3 different unaffected donors	Ctr1_LCL_H33	Ctr2_LCL_H33	Ctr3_LCL_H33				
**LCLs**	H3.3 ChIP-seq case	3	3 different patients	Case1_LCL_H33	Case2_LCL_H33	Case3_LCL_H33				
**LCLs**	H3K27me3 ChIP-seq control	3	3 different unaffected donors	Ctr1_LCL_H3K27me3	Ctr2_LCL_H3K27me3	Ctr3_LCL_H3K27me3				
**LCLs**	H3K27ac ChIP-seq control	3	2 different unaffected donors (see note)	Ctr1_LCL_H3K27ac	Ctr2_LCL_H3K27ac	Ctr1_rep2_LCL_H3K27ac				Ctr1_rep2_LCL_H3K27ac was performed independently to assess the replication of Ctr1_LCL_H3K27ac experiment but it is not included in the average representation of the control
**LCLs**	H3K27ac ChIP-seq case	3	2 different patients (see note)	Case1_LCL_H3K27ac	Case2_LCL_H3K27ac	Case1_rep2_LCL_H3K27ac				Case1_rep2_LCL_H3K4me3 was performed independently to assess the replication of Case1_rep2_LCL_H3K4me3 experiment but it is not included in the average representation of the cases
**LCLs**	ATAC-seq performed on gDNA control	2	2 different unaffected donors	Ctr1_gDNA_LCL_ATAC	Ctr3_gDNA_LCL_ATAC					
**LCLs**	ATAC-seq performed on gDNA case	2	2 different patients	Case1_gDNA_LCL_ATAC	Case3_gDNA_LCL_ATAC					
**LCLs**	ATAC-seq control	4	4 different unaffected donors	Ctr1_LCL_ATAC	Ctr2_LCL_ATAC	Ctr3_LCL_ATAC	Ctr4_LCL_ATAC			
**LCLs**	ATAC-seq case	4	4 different patients	Case1_LCL_ATAC	Case2_LCL_ATAC	Case3_LCL_ATAC	Case4_LCL_ATAC			
**LCLs**	Bio-CAP-seq control	3	3 different unaffected donors	Ctr1_LCL_BioCAP	Ctr2_LCL_BioCAP	Ctr3_LCL_BioCAP				
**LCLs**	Bio-CAP-seq case	3	3 different patients	Case1_LCL_BioCAP	Case2_LCL_BioCAP	Case3_LCL_BioCAP				
**LCLs**	Capture-C control	3	3 different unaffected donors	Ctr1_LCL_CapC	Ctr2_LCL_CapC	Ctr3_LCL_CapC				
**LCLs**	Capture-C case	3	3 different patients	Case1_LCL_CapC	Case2_LCL_CapC	Case3_LCL_CapC				
**Erythroblasts**	H3.3 ChIP-seq control	2	2 different unaffected donors	Ctr1_Ery_H33	Ctr4_Ery_H33					

**Erythroblasts**	H3K27me3 ChIP-seq control	1	1 unaffected donor	Ctr4_Ery_H3K27me3						
**Erythroblasts**	H3K4me3 ChIP-seq control	1	1 unaffected donor	Ctr1_Ery_H3K4me3						
**Erythroblasts**	H3K4me1 ChIP-seq control	1	1 unaffected donor	Ctr1_Ery_H3K4me1						
**Erythroblasts**	H3K27ac ChIP-seq control	3	3 different unaffected donors	Ctr1_Ery_H3K27ac	Ctr2_Ery_H3K27ac	Ctr3_Ery_H3K27ac				Ctr1_Ery_H3K27ac was normalised per 100 million reads and used in Figure 3 . Ctr2_Ery_H3K27ac and Ctr3_Ery_H3K27ac were performed as ChIP-Rx and normalised based on the Drosophila melanogaster S2 cells spiked in and used in Figure 5.
**Erythroblasts**	H3K27ac ChIP-seq case	2	2 different patients	Case1_Ery_H3K27ac	Case2_Ery_H3K27ac					Case1_Ery_H3K27ac and Case2_Ery_H3K27ac were performed as ChIP-Rx and normalised based on the Drosophila melanogaster S2 cells spiked in and used in Figure 5.
**Erythroblasts**	input control (one step cross linking)	3	3 different unaffected donors	Ctr2_Ery_InputSC	Ctr3_Ery_InputSC	Ctr1_Ery_InputSC				
**Erythroblasts**	input case (one step cross linking)	2	2 different patients	Case1_Ery_InputSC	Case2_Ery_InputSC					
**Erythroblasts**	ATRX ChIP-seq control	4	3 different unaffected donors (Ctr1_rep1_Ery_ATRX and Ctr1_rep2_Ery_ATRX are two replicates from the same donor)	Ctr1_rep1_Ery_ATRX (Ctr1_Ery_ATRX)	Ctr1_rep2_Ery_ATRX (Ctr12_Ery_ATRX)	Ctr2_Ery_ATRX	Ctr4_Ery_ATRX			
**Erythroblasts**	input control (two steps cross linking)	3	3 different unaffected donors	Ctr1_Ery_InputDC	Ctr2_Ery_InputDC	Ctr4_Ery_InputDC				
**Erythroblasts**	GATA1 ChIP-seq control	2	2 different unaffected donors	Ctr2_Ery_GATA1	Ctr3_Ery_GATA1					
**Erythroblasts**	GATA1 ChIP-seq case	2	2 different patients	Case1_Ery_GATA1	Case2_Ery_GATA1					
**Erythroblasts**	ATAC-seq control	6	3 different unaffected donors (Don002_Rep1_Ery_ATAC, Don002_Rep2_Ery_ATAC and Ctr2_Ery_ATAC are three replicates from the same donor and Ctr1_Rep1_Ery_ATAC and Ctr1_Rep2_Ery_ATAC are two replicates from the same donor)	Ctr1_Rep1_Ery_ATAC	Ctr1_Rep2_Ery_ATAC	Don002_Rep1_Ery_ATAC	Don002_Rep2_Ery_ATAC	Ctr2_Ery_ATAC	Ctr3_Ery_ATAC	Don002 and Ctr2 are samples derived from the same unaffected individual. However, the names are different to differentiate between Ctr2 ATAC and H3K27ac experiments that were performed in parallel with Ctr3, case1 and case2 ATAC and H3K27ac experiments used in Figure 5 and don002 ATAC experiments that were performed independently and used in Figure 3 with Ctr1 ATAC. Samples from Ctr1 and Don002 were used for peak calling.
**Erythroblasts**	ATAC-seq case	2	2 different patients	Case1_Ery_ATAC	Case2_Ery_ATAC					
**Erythroblasts**	Capture-C control	2	2 different unaffected donors	Capture_Ery_Control2	Capture_Ery_Control3					
**Erythroblasts**	Capture-C case	2	2 different patients	Capture_Ery_ATRX_Case1	Capture_Ery_ATRX_Case2					
**Erythroblasts**	scRNA-seq control	1	1 unaffected donor	Ctr3_Ery_scRNA						
**Erythroblasts**	scRNA-seq case	1	1 patient	Case1_Ery_scRNA						
**Erythroblasts**	scATAC-seq control	2	2 different unaffected donors	Ctr2_Ery_scATAC	Ctr3_Ery_scATAC					
**Erythroblasts**	scATAC-seq case	2	2 different patients	Case2_Ery_scATAC	Case1_Ery_scATAC					

Supplementary Table 4

Summary of the experimental replicates for high-throughput sequencing data and associated comments.

chr1:15480096-15480166_1	GATCCCTCGCCTCGGGCCCGGGGGTGGTGCCGAGGGCCGGGAGGGAGGGTCGGCCGGTGGGCGGTGCTTC
chr1:15480278-15480348_1	GAGGGCAGTGCGCCCTCTCCACCACTGCGTTCCCTCGGCTAAGAATCCCCCGAACCCCAGCCCCGCGATC
chr1:24195399-24195469_3	GATCACTTGGGAGCCATAGGGGGCTTTTGAGTTGTCCTGTAGCACAAGGCTGGAGGAGGAGATAACAGAC
chr1:38259380-38259450_4	GATCGACGAAGAAGGAAATCCTTGGGGAGAGAACCACCGACGCGCGCTTCTTTCCCCCCCCCGCCCCGCC
chr1:38260195-38260265_4	GAGCCCGCGGCGCGAGGGCCACTACATTCACTGGGACCACGTCATGGTGCCGCACTGGGACCCCAAGATC
chr1:109204333-109204403_6	CGCCCCCTCTCCACCGCCACCCGACCCCCGAAATAGTTTTCCCCGAGTGTTGTGGATAGAACTGTTGATC
chr1:154831423-154831493_10	GATCTTGCCCACCCAGGCTAGCACACCTGGCTGGCCTACAGAGCTTGGCCATCTAGGGCAGGTATGGTGC
chr1:154841922-154841992_12	GATCTCCGTGAAGGGGTTGCTGTCCCGCCGGTGCACCAGGGGGCTGGCCTGTCGGTGCCGGCTGCCTCCG
chr1:154843227-154843297_12	TCTCATTTACAAAGCCTGATGCTTAGAGGTGTTGAGTAACAACGTCAAGGTCACAGAAGCAGGGGGGATC
chr1:158901458-158901528_13	AGTTTCTGAGAGCTTTACTGACTGATTTCCCTATTCAAAACAATCCTCATTTCCTACATTTCTGAAGATC
chr1:145438534-145438604_7	GATCTTTTCTCCAGCAATTGGGGGAAAGAAGGCTTTTTCTCTGACTTCGCTTAGTGTAACCAGCGGCGTA
chr1:145438753-145438823_7	TTTTTCTTCCACCGTCATTTCTAACTCTTAAAACCAACTCAGTTCCATCATGGTGATGTTCAAGAAGATC
chr10:43722502-43722572_16	GATCAGCTTCCAAGGATAACCAGGGGTTTTCCAGTGGGTTAAGGGGGAAAAGGGAGGTTGACATGACAGT
chr10:43725415-43725485_16	GCGCGCCTGGCGCTAGGTGTGGGGGCGCCGTAGGTGCCGGGGTGTGAGGCTGGACGGGGCGTCCGGGATC
chr12:6643414-6643484_18	GATCACCTCCCATCGGGCCAATCTCAGTCCCTTCCCCCCTACGTCGGGGCCCACACGCTCGGTGCGTGCC
chr12:6644223-6644293_18	GGCGCCATCTGCCCGGAGCCTCCTTCCCCTAGTCCCCAGAAACAGGAGGTCCCTACTCCCGCCCGAGATC
chr12:10517803-10517873_3	GATCCTTCCTACCTATATTCCAATCTATCCCCCCTTACCAAGGCTAATTGCCCCTAAGGAGGTAAGTTCA
chr12:117198844-117198914_20	GATCCCTGAATGTTCCTTCTTTCTCCCCTCCAACAGCAGCATCTCCTCCACGTACACCTAGCCCAGTCCC
chr13:49000503-49000573_22	GATCATGTGATGCATTTGGTATGCCTTTTTTAGCACTGCTAATGTACAGCCAATTTAAATCAAATAGACA
chr13:49000615-49000685_22	GCTTGGTGGTTAACACATTGCATATTGCATTAGTCTTAATATCCTCTAATGTTGGAGCTAGCACTGGATC
chr13:49019550-49019620_24	GATCATACAGCAACCTCTTCTTTAGCAACCTCTCCACTATCCCACCCGGGTACTTTAGTTCTGGTGGCCT
chr14:23284600-23284670_25	GATCTGTGGTCTGATGCTCCTCCTTCCCAGCCAGCAGTAAAAGGGAAGGCCAGACAAATGCCTATGGATT
chr14:23285201-23285271_25	CTCCAAGCCTGGGCCACTGCCCCAAAGTCTGAGCCTCAGACTACTTCAAAAGAAACACAGTCAAGAGATC
chr14:23288739-23288809_26	GATCAAGAAACAGGAGGCTCTTGGGTCTCCATTCAGCCAATTTTTAGGACTCCATCCTCCTCCTGCACTT
chr14:23289275-23289345_26	TCCACTCATCCCATTTCCTACTCATCTCCCACTCATGTACTATCCTTAGTTCTGCAGAGGGGATACGATC
chr14:50469891-50469961_27	GATCTGCAACTCAAAGTCAAGGGATTCCGTGTCCCTGCTTGTGTCAACATAGAAAACGCCCATGGCAACG
chr15:37393257-37393327_28	AAGCTCTGCAGACGGTACCACTGCCGGGCTAAATTTTTTATTAAGTGGCACTTTCTTTTCTGCGCAGATC
chr15:75249070-75249140_31	GATCTCGGCCCCACTTCTCTCGAGCGGGCGCCGCTCTACTCAGCGCTCTCGGCGCCGGGTGCTCCGCGCC
chr15:75249879-75249949_31	GCCTTGCCAACGTGGCCGCCCCCTTCCTCCCAGGCGGAATTCGCTCAGGCAACATTGGCCCCTTGAGATC
chr15:99646569-99646639_33	GATCTAAGAGTCAACCGAGAAACCTCTCTCACTTGCAGAAGTGACGGCTTATGGTAGCCCCGCTGTCTAT
chr15:99646683-99646753_33	TATTTTGCCAATATGATTTCCCAGTTGGAAGCCGCAGTTGTGGCTCTCTGGGCGGCTGCCTTGTTTGATC
chr16:447865-447935_35	GATCCATGTCCCAGGGCTCCCTCCATCGGCTCTGAAAAGTGAGCCGCCGCCTGCAATGCCCGCACCAGCC
chr16:448476-448546_35	GGCTTCAGACCATGTCTGTCGTCATCCAGAAGGGATGGAGTCAAAAGTGAGGCTGGAGTTTTGCAGGATC
chr16:4587755-4587825_36	GATCTTGAGCCCACCTTTGGAGGAACTCACAGCCACTCGCCCCACCTCATCTAACACCTGTTGAGAAGCC
chr16:10480384-10480454_37	TCCCATTGCCAAATTTTCTTGAGTTGTTTTAATCATCTAATGAAGTCAGGTGCCAGAAGGCAACTGGATC
chr17:38024391-38024461_39	GATCGCCTGCAGCGCCGTCGCCCCGCCCCCGCGCGCGAGAATCGAAGCACGCACGCGTTCTCCTGCGCGT

chr17:38024971-38025041_39	GAGCATCTATTATACTAAGCATCTGCTTGGCAGTTCACGACGGTCGCATTTTTTCATCCTTACAGCGATC
chr17:67111137-67111207_40	GATCTCATTTAAGAAAATATTGGCATATGTTCACAAATGTCTAAAATGATTTGTGAACTACTATTCTTGA
chr17:67111700-67111770_40	CATAGAACAAGGCTATTTAGTTTAGTGGCATATTTCTCAGCATTTCAATACTTTAAAATACACAATGATC
chr18:6413933-6414003_42	GATCAAAAGCAGGGGACAATACCCACCCGGAATTTGCGACCTCGCGAAAACGCTCTCACCAATCCCGCAG
chr18:6415187-6415257_42	TCCCAGTAGCTCACCCATGCTTCATCCCAGCTCTTGGGGTTCACCGTCTGAAGGCTCTCTCCTCCGGATC
chr19:2841594-2841664_44	GATCCCGGTGCGGGGCAGCAGGAACCGGCGACCGCCCGAGACCGCGGCTTGGGGGGAGCTGAGGGACGCG
chr19:2843080-2843150_44	AAAAATTAAAATCTGTGTCTTACATTCCATTTGTTAAACATTCCCTGGAGTGTGTGTCGTTTCTCAGATC
chr19:2899877-2899947_46	GATCAGGTGACTCGAGGTTTGGTCCTTATTTTAGTGCAGGCTCAGATGTCGGGGGCGAGCCAAAGTGCAG
chr19:6887676-6887746_48	AGGGGAGGCCTGGATTGGAAATAGACTTCAGGAGAAATGGGAGGGCCATGGATGGTCCAGCCAAGAGATC
chr19:19708967-19709037_50	GATCATTGTACTCAGCGTAATTTCCTGGTTTCCATATTTGTACTATGGCTCTGAAGGTGTTGACGAGACA
chr19:19932534-19932604_51	GATCTCTGGCGTCAGCGAGAGACAATGGGCCCGCCAAAGCCGGAAGCCGGAAACCGTCCTGTTCGCTACA
chr19:49990649-49990719_52	GATCTCTCGCCGGGCACAGCGCTGACCGCGGAGGTCCAACCGGAAGAATGTCCGGATTGGACATTCGGAA
chr19:49990877-49990947_52	CTGCCGAAGATGGCGGAGGTGCAGGTATGGGCTCCGCGCGGGCCGGGGCGGCAAGGGGCCGGGTGGGATC
chr19:51377673-51377743_53	ATCACTCCAAATGACCCAACCCCCAACCCGATGCCTGCTTCAGATGTTTCCCATGTCCCCTACTCTGATC
chr19:55174488-55174558_57	GATCCCCACCTTCACGGCTCTGCTCTGCCTCGGTGAGATTTAAAGAGGGGGAGGGGAGACCCGAGTCTTG
chr2:30453858-30453928_58	GATCGCAAGGCTGCGGGCGAGGATTCCAGAGAGAGGCCTAGTATGGGGAACAAACGCTTCAGAGGGGTCC
chr2:74780377-74780447_60	GATCCCCTGAATGGGAGCCCAGAGTCCTAACCCAGCAGATTCCCAGGCAGGGAAGGCTGGCACCAAAGCT
chr2:74780985-74781055_60	AGCGTGACTCCCCCTACCTTGGCCGAGCAGGACCCTAAGGGCTGGGAGAAGAGTGCTGGTCCTGGAGATC
chr2:96700545-96700615_62	GATCCCGGTCCCCTTCTGACCTGGCCTCGCCGGGGAGCGACCACCGCGCCTCTCACCTCACACCAGCTTC
chr2:96700952-96701022_62	CGCTCCTCCAAGCTGGGGTTCGGCTCTAGCAAAATTCTGAGCTGGGGACGATTAGAAAGGAGAGAAGATC
chr2:99347829-99347899_63	GCGGTCTCGGGTCCCAGCTCTGGGCGCCGGCCTGCTCCCCGCCGCCCCGCTGCCACATCTGGCTGAGATC
chr2:192015140-192015210_64	GATCCCACTAAAGGGGCATTTTGTCCCACCGTCTGTGAGCTGGTGGTGCCGCTTTGGCTCTGTGGCCTGT
chr2:192015971-192016041_64	CAGTGCCAGGAAAAAGGGGAGGGGCGGGGCATACATTTTCTTCAGTGGTTTCCTTAGACAATCTGAGATC
chr2:204571203-204571273_67	CATCAAAACAACGTTATATCCTGTGTGAAATGCTGCAGTCAGGATGCCTTGTGGTTTGAGTGCCTTGATC
chr20:62679722-62679792_70	GATCGGCGGCGGGCCCCAGCGGCTCGGCGCTCTCCAGCGGCGGGGCCTCGGGCGGCGGCGACAGCGGGCC
chr20:62680792-62680862_70	TGTCCCGGGGCCCATGCACAGTCGCGGCGGGCGGGCGGGTCGTCCTGTGCGCCGTAGCCGGGCGGCGATC
chr20:61488669-61488739_69	GATCACTCATCTGTGATAACCATGTATGACTAAGGTAATCATATATGAGTCAGCCTGACAAGCAGTCACA
chr22:36649139-36649209_71	TCCTCGGTATATCTTGGGGACTGGAGGACCTGTCTGGTTATTATACAGACGCATAACTGGAGGTGGGATC
chr3:46249848-46249918_74	GATCCTGACCAAGTAGGAAGTACTCAGCTTCCTGTGTGATGATGGAGAATTGGCTCCAGTTTCACAGTCT
chr3:112693421-112693491_75	GATCTGTATATTGTAGTAGGATACAAAGTACAACAAGCAAGACACCGGAAAAAAGGATAATAAAGTAAAC
chr3:112693744-112693814_75	CTCCACACAGGTACAGAAGGAACTGTGCGCATGGTGAGACCCTCTCTGGTCAACTTCTCAGTACAGGATC
chr3:122043239-122043309_77	GATCTATGTGTATATCAACATAGAAAGGCTCAAAAACATGTTGAATAGAAAAAGGAACATAACATAGAAT
chr3:122043606-122043676_77	TGTTAAACATTTCCAGATATTTAAATAAGAGTAAAGAAGACACATCCAGCCAAGGTCCTCCAGATAGATC
chr3:138312871-138312941_79	GATCCCCAAACCCTTACGCAGGGATTTTTAATTGGCGCGACCCTGTCCCAGATGGCTCCTCCCCGACAAG
chr3:138313026-138313096_79	GAGAGCCACTCACCTTACTCAGTCGCCGGCCTCCCACCAGCAGCCAGCCGGGCCAGGTTAGGCTGGGATC
chr3:141121383-141121453_81	GATCGCTCCTCGCTTCTGGAGGGAGCCGGGAAGCCGGGCTGGGGAAACCCTCGCAAGGGTTGAGCTCTGC

chr3:141121982-141122052_81	GCACTGCGGTAGGTAAGTGCCTAATTCCATTCTGGACGTTGGCCCTAATTGAGTATCCCTTCTAAGGATC
chr3:141105288-141105358_16	GATCATGCATTTTTCTACAAGCCTGCCGGCACCATTTAGGATTTTGAAAAATAATCTAGAAATGCTATAC
chr3:141106496-141106566_16	AGAAAAACCTTGGATGAGAAACTTCTTTTTTCAATCCTGTTTTCCTGTTGATAAATTCAGTTCTGTGATC
chr3:61547232-61547302_23	GATCCATGCTCACATGTTACTTCCTGTATGGAGGCATGGCCAGTTTCCAGCCCCGCGCTCTTCGTTCCTT
chr3:61547306-61547376_23	CCTGCGCCGGAGCCACAACTTTCAGGAGCATGGACTGAAGGCGCCCTCGCCCCAGCGCCCCTCTGAGATC
chr4:124373-124443_83	GATCTGGCGCGGCTTTTGCTTGTAGCTCCAGCCAGAGCTCGGTTAGGGCCTCATCGCTCTGCTCCCGCTC
chr4:619185-619255_84	GATCCCCCAACAGTTACTCCCAGCCTTCATTCCACAGGGTCTGGTTTTCCTGGAAGGTGGGAAGTCCCAG
chr4:619735-619805_84	CACCAGGCTTTTCAGCGTGCAGCCGGACAGCGTCCTGGAGGACTGCCTGGTGCCCCCCGACTCCGAGATC
chr4:647333-647403_85	TGGTCGGATGTTTGCCTGTCAAGCTCAAAGCGACAGATTCCACTTTCCACTCGTCCACTCCTGAGTGATC
chr4:187644579-187644649_3	GATCGGTCCCGCGCGGTCGCCTCCTTTTCCCGCTCCGCCGCCGGGACCCCCCTAGCCAGTGCTGAATTTC
chr4:187647623-187647693_6	GATCTTCAGCATGTCCCTAACCAGAGTGCCAACGTCCGTGCCGCCGGGACCCAGGCGGCCGCATCTCCGG
chr4:187648735-187648805_6	TGAAATTCCGAGGGAGCCATTTCTGTGAGTTGACATTGTCCCCAGAAGATTGTCATTAACATTCAAGATC
chr5:43192722-43192792_87	GATCCGCAAGCGAGAGGAGGGGGCATTAGAGCCAGGGCGAGAGCGTGCTCGCGCGTGTGTGCCCGTGTCA
chr5:43192947-43193017_87	TGGACCAGAGCCCTAAGGCACCAGACCAAATCTGGAGCGTTTGTTACAAGGGCAGAAGACCGGCGTGATC
chr5:95066371-95066441_22	GATCACACTAAAAATCCCAGCATTTAAAATCCTGAAGTTTTCGGTAAAATACGCTAAATATAGCTTGAAA
chr5:95066920-95066990_22	CCGCATCCGCCCGACCCCCGGGGCTGGCACGTGCTGCGCCCGGTCCGCTGAGGGGGCGGAGGCCCCGATC
chr6:26017820-26017890_89	GATCAGCTCTGACACGGAAGGGCCAGCGGGTTTTTTCTTGGAGGCTGCTGCAGCCTTAGCAGGTTTCTTT
chr6:26018969-26019039_89	ATGGTAACAACCCTTTAACTTGTTTGTGGCTGTTAAGTGCATTTCAAAGTATCAATAAGCTAAAACGATC
chr6:31582624-31582694_90	GATCTACAGAGCAGAAAAATTCAGCCAGCCCTTCCTTGTCTTCCTATCCACAGCTGCCTGCCCAGACTCA
chr6:31583095-31583165_90	TCTGCTGAGCTATGAGCCAAACCAGGGATTTACAGGGTAGGGAGGGTGGGATAGGCAGCGGCATTAGATC
chr6:135818320-135818390_91	GATCACCTTTTCTCAGACATAATTAAGAAGGCTTTGACAGGTTGAAGATTCAGCCCCAGGTTGACACTCT
chr6:149067356-149067426_92	GATCTATGTCTAGGATTGAGCCTTTTCTGCCCCAGGAGGTTCTTTCAGGTCCAGAGGCTTTCCCGTGGAG
chr6:149068536-149068606_92	GGCAGGCTGTGGGAGGCAGCGGAGCAGGCGATGAAGAAGAAGCAGCAGCATCCCGGCGGCGGCGCGGATC
chr6:149639183-149639253_94	GATCCCCAACCTGGCTCTCCCCTCATCGCCCCGCACCCCCCTCCGTCTGGAAGGGGCTCTGCCGCCTCGC
chr6:149639607-149639677_94	TTTTGGGGACAGACACGGGGCAGTTATGTGGGGGTCGGGCGGGAGGGAGAACATCGTTCCTTTGCGGATC
chr7:76828658-76828728_96	GATCTCCTCTTTGGCATTCTTTAGCTCAGAGGACAGCTTGTTAACCTCACTCTCTAATTCTCTAACTCTG
chr7:76828819-76828889_96	TTGGCAAGATTTCTTTAGACTATTTACGATTTCCTTGAGGTTTTGGACTTCTTTGAACACCTCCTCGATC
chr7:98029298-98029368_97	GATCCTAATTACAGGGGAGTTTCACCCCCACATTCTTGGGCGCTTTCTTTTCTTAGAGGAAACTCGAGCT
chr7:98030850-98030920_97	CAAGCCCTGCGTTTTGGGGACCAAGGGAAGGGGCCACATTCCCCAGTTGTATTGATTTCCCTGATTGATC
chr7:76751285-76751355_95	GATCCGGGCCAGCGCTCGCCCCTACCTGAGCTGTACGGCCTGGCTTGGCAAGCCCAGCAAGACCAGCGCC
chr8:2075166-2075236_98	GATCTCTGCCTTACAGATTTTCAATAACCCTCCTGTCTGCAGCCGGCCCAGATGCAGGGACCCTCCAAGT
chr8:2076291-2076361_98	CTTCCCAAAATGAAAAAATACTATTTAAAATGCCTCTTTGTTACACTATCCTCCACAACAGTTGGTGATC
chr9:74525635-74525705_99	GATCCGCGCTCCGGGCGCCGTCCGACTCTCTCCGCCTGCAGACTGGCCCGACAGAGGAGCGGGGACCAGG
chr9:74526985-74527055_99	TGAACAGGACCCTAATGAAATCTAAGGTGCAGATGGGGCAGTTACGTCGTTCAGCGTTCCCATCTGGATC
chr9:74764173-74764243_100	GATCTTGATAACTCAAAGAGTTAACACAACCAAAACTAACGCAAGGGTGGAGAGAAAGACCACACCCCAC
chr9:74764499-74764569_100	CTGGCGCACATCTTCCGAGGGACGTTCGTCCACTCCACCTGGACCTGCCCCATGGAGGTGCTGCGGGATC

chrX:41547752-41547822_101	GATCAAAAGGGGAGAACACCATCAGAAATCTCATCTAGTCCTAATAATACAAGTTTGTTTTAGCAAAGGT
chrX:41548568-41548638_101	AGCTCCTACTGTCAATATAGTCACATTGTTGATACTGCATTTGGATACTGCCATCAAGATTTTGCAGATC
Hba-1_5p	GATCCCGCTGGAGTCGATGCGCGTCCAGCGCGTGCCAGGCCGGGGCGGGGGTGCGGGCTGACTTTCTCCC
Hba-1_3p	CGACCTGCACGCGCACAAGCTTCGGGTGGACCCGGTCAACTTCAAGGTGAGCGGCGGGCCGGGAGCGATC
Hba-2_5p	GATCCCGCTGGAGTCGATGCGCGTCCAGCGCGTGCCAGGCCGGGGCGGGGGTGCGGGCTGACTTTCTCCC
Hba-2_3p	CGACCTGCACGCGCACAAGCTTCGGGTGGACCCGGTCAACTTCAAGGTGAGCGGCGGGCCGGGAGCGATC
Hbb_5p	GATCCCCAAAGGACTCAAAGAACCTCTGGGTCCAAGGGTAGACCACCAGCAGCCTAAGGGTGGGAAAATA
Hbb_3p	AAAACATCCTCCTTTGCAAGTGTATTTACGTAATATTTGGAATCACAGCTTGGTAAGCATATTGAAGATC
Myc_5p	GATCCTCTCTCGCTAATCTCCGCCCACCGGCCCTTTATAATGCGAGGGTCTGGACGGCTGAGGACCCCCG
Myc_3p	CTCCCCTCCTGCCTCGAGAAGGGCAGGGCTTCTCAGAGGCTTGGCGGGAAAAAGAACGGAGGGAGGGATC
Slc25a37_5p	GATCAGGCCTCTACAGACCTTATGGGCCTATCAAACCCAAAGAAAATTTCCTGGAGGTGCCTACTCAGAG
Slc25a37_3p	GGACTACGAGAACCTGCCGACTAGCGCCTCCGTGTCCACCCACATGACAGCAGGAGCGATGGCCGGGATC
Hbd	GATCCCCAAAGGACTCAAAGAACCTCTGGGTCCAAGGGTAGACCACCAGTAATCTGAGGGTAGGAAAACA

Supplementary Table 5

List of Capture-C oligos
