# Supplementary material for: The chromatin remodeller ATRX facilitates diverse nuclear processes, in a stochastic manner, in both heterochromatin and euchromatin
Source: Nat Commun. 2022 Jun 17;13:3485. doi: 10.1038/s41467-022-31194-7 (PMC9203812; doi:10.1038/s41467-022-31194-7)
Supplement: Supplementary file 3 — Reporting Summary [file 41467_2022_31194_MOESM3_ESM.pdf]

nature portfolio

Corresponding author(s): Gibbons RJ & Higgs DR

Last updated by author(s): Apr 25, 2022

Reporting Summary

Nature Portfolio wishes to improve the reproducibility of the work that we publish. This form provides structure for consistency and transparency in reporting. For further information on Nature Portfolio policies, see our Editorial Policies and the Editorial Policy Checklist.

Statistics

For all statistical analyses, confirm that the following items are present in the figure legend, table legend, main text, or Methods section.

n/a	Confirmed	
☐	☒	The exact sample size (*n*) for each experimental group/condition, given as a discrete number and unit of measurement
☐	☒	A statement on whether measurements were taken from distinct samples or whether the same sample was measured repeatedly
☐	☒	The statistical test(s) used AND whether they are one- or two-sided<br>*Only common tests should be described solely by name; describe more complex techniques in the Methods section.*
☐	☒	A description of all covariates tested
☐	☒	A description of any assumptions or corrections, such as tests of normality and adjustment for multiple comparisons
☐	☒	A full description of the statistical parameters including central tendency (e.g. means) or other basic estimates (e.g. regression coefficient) AND variation (e.g. standard deviation) or associated estimates of uncertainty (e.g. confidence intervals)
☐	☒	For null hypothesis testing, the test statistic (e.g. F, t, r) with confidence intervals, effect sizes, degrees of freedom and P value noted<br>*Give P values as exact values whenever suitable.*
☒	☐	For Bayesian analysis, information on the choice of priors and Markov chain Monte Carlo settings
☒	☐	For hierarchical and complex designs, identification of the appropriate level for tests and full reporting of outcomes
☒	☐	Estimates of effect sizes (e.g. Cohen's *d*, Pearson's *r*), indicating how they were calculated

Our web collection on statistics for biologists contains articles on many of the points above.

Software and code

Policy information about availability of computer code

Data collection	none
Data analysis	Several previously published software packages were used for the analysis of the data. These included FASTQC 0.11.9, Bowtie 2.3.2, Samtools 0.1.19, Bedtools 2.25.0 to 2.29.2, Deeptools 2.2.2 to 2.4.2, ucsctools 373, MACS2 (Zhang et al., 2008) and HOMER 4.6 to 4.11 (Heinz et al., 2010). ChIP-seq, Bio-CAP-seq and ATAC-seq data were analysed using a in-house pipeine (github: https://github.com/Hughes-Genome-Group/NGseqBasic/releases) (Telenius & Hughes, 2018). Data were visualised on the UCSC genome browser (GRCh37/hg19) (http://genome.ucsc.edu/). Capture-C probe design and analysis wer performed using CapSequm v1 (http://apps.molbiol.ox.ac.uk/CaptureC/cgi-bin/CapSequm.cgi) and in-house pipelines CCseqBasic VS1 (Telenius, 2020) (GitHub : https://doi.org/10.5281/zenodo.4196777). Microarray data were analysed with Affymetrix® Transcriptome Analysis Console (TAC) and Affymetrix® Expression Console™ Softwares Affymetrix and the R packages limma (Ritchie et al., 2015) 3.48.3, affy 1.70.0 (Gautier et al., 2004) and genefilter 1.74.1 (Gentleman et al., 2016). Gene onthology was performed with GOTERM_BP_DIRECT DAVID 6.8. Single cells data were analysed with cellranger/3.0.2, cellranger-atac/1.1.0 and R packages Seurat 3.2.0 (Stuart et al., 2019), Signac 1.0.0 (Stuart et al., 2020), cisTopic v3 (González-Blas et al., 2019) and ggplot 2 3.3.2. Statistical analyses were performed using GraphPad Prism 9 or R 3.6.1.

For manuscripts utilizing custom algorithms or software that are central to the research but not yet described in published literature, software must be made available to editors and reviewers. We strongly encourage code deposition in a community repository (e.g. GitHub). See the Nature Portfolio guidelines for submitting code & software for further information.

Data

Policy information about availability of data

All manuscripts must include a data availability statement. This statement should provide the following information, where applicable:

- Accession codes, unique identifiers, or web links for publicly available datasets
- A description of any restrictions on data availability
- For clinical datasets or third party data, please ensure that the statement adheres to our policy

The microarray and sequencing data generated for this study have been deposited at Gene Expression Omnibus (GEO) under the accession numbers : GSE192767, GSE193038, GSE193310, GSE193311, GSE193312, GSE193314 and GSE193315. Additional data used in this study are available under the GEO accession numbers : GSM733752, GSM758559 and GSE125924 64 as well as in the Uniform TFBS (wgEncodeAwgTfbsUniform) dataset associated with LCLs. Genome assemblies : Homo sapiens (human) genome assembly NCBI36 (hg18) (https://www.ncbi.nlm.nih.gov/assembly/GCF_000001405.12/), Homo sapiens (human) genome assembly GRCh37 (hg19) (https://www.ncbi.nlm.nih.gov/assembly/GCF_000001405.13/).

Field-specific reporting

Please select the one below that is the best fit for your research. If you are not sure, read the appropriate sections before making your selection.

☒ Life sciences ☐ Behavioural & social sciences ☐ Ecological, evolutionary & environmental sciences

For a reference copy of the document with all sections, see nature.com/documents/nr-reporting-summary-flat.pdf

Life sciences study design

All studies must disclose on these points even when the disclosure is negative.

Sample size	For the microarray experiment, sample size was determined based on power calculation using ssize package. A sample size of n=20 per group (control and case) was sufficient to detect 99% of the probes with a fold change of 1.5. We used n=20 for the control group and n=28 for the patient group (knowing that a same ATRX mutation can results in variability in phenotypes between patients).<br>No statistical method was used to predetermine sample size for the other experiments. Experiments were performed in 2 to 6 independent replicates for Bio-CAP-seq, ATAC-seq, Capture-C and ChIP-seq as is common in the field (Valle-García et al.,2016, Godfrey et al., 2019 and Bozhilov et al., 2021) at the exception of the erythroblast ChIP-seq samples H3K4me1, H3K4me3 and H3K27me3 which were performed in only one independent replicate due to the prioritisation for the use of limited material. Erythroblast ChIP-seq samples for H3K4me1 and H3K4me3 corroborated published data at the alpha-globin cluster (Bozhilov et al., 2021). scRNA-seq was only performed with 1 independent replicate per group due to sample availability but the analysis was performed on 4,400 cells per sample and the results were in line with the patient phenotype as discussed in the manuscript. The number of replicates is specified in a summary table in supplemental information.
Data exclusions	none
Replication	Microarray data were validated using qRT-PCR experiment on 20/21 candidate genes including 17/18 DEGs.<br>ATRX ChIP-seq in LCLs were performed with two different antibodies showing similar results (only peaks detected by both antibodies were kept in the final peak calling including all the samples).<br>For other experiments, replicates from samples derived from the same individual (unaffected donor or ATR-X case) gave similar results, replicates from samples derived from different unaffected individuals gave similar results and replicates from samples derived from different patients reflect the heterogeneity observed in the phenotypes knowing that even siblings with the same ATRX mutation can show variability in the severity of their phenotype as discussed in the manuscript. This is exemplified in Figure 5 by experiments using erythroblasts derived from two siblings having variable degrees of alpha-thalassaemia (case1 with alpha thalassaemia and case2 which has not been diagnosed with alpha thalassaemia (absence of HbH inclusion)). scRNA could not be replicated due to limited sample material. Erythroblast ChIP-seq samples H3K4me1, H3K4me3 and H3K27me3 were not replicated due to prioritisation of the material available. However, ChIP-seq samples for H3K4me1 and H3K4me3 corroborated published data at the alpha-globin cluster (Bozhilov et al., 2021).
Randomization	Experiments were not randomized. Experimental groups of cells were selected by genotype or patient phenotype.
Blinding	Investigators were not blinded during experiments or analysis. Blinding was not relevant to this study as the data presented are objectively obtained by quantification of cellular phenotypes through measurements of such as mRNA levels and chromatin interactions and all samples from compared groups were analyzed using the same pipelines and/or scripts. Experimental groups of cells were selected by genotype.

Reporting for specific materials, systems and methods

We require information from authors about some types of materials, experimental systems and methods used in many studies. Here, indicate whether each material, system or method listed is relevant to your study. If you are not sure if a list item applies to your research, read the appropriate section before selecting a response.

Materials & experimental systems

n/a	Involved in the study
[ ]	[x] Antibodies
[ ]	[x] Eukaryotic cell lines
[x]	[ ] Palaeontology and archaeology
[x]	[ ] Animals and other organisms
[ ]	[x] Human research participants
[x]	[ ] Clinical data
[x]	[ ] Dual use research of concern

Methods

n/a	Involved in the study
[ ]	[x] ChIP-seq
[ ]	[x] Flow cytometry
[x]	[ ] MRI-based neuroimaging

Antibodies

Antibodies used

CD235a GPA PE BD Bioscience Cat no 555570
CD71 Transferrin PerCP Cy5.5 Biolegend cat no 334114
CD49D α-Integrin APC BD Bioscience 5Cat no 61892
CD34 PE/Cy7 Biolegend Cat no 343616
CD233 Band3 FITC IBGRL Cat No 9439FI
CD36 Platelet glycoprotein APC/Cy7 Biolegend Cat No 336213
Hoechst 33258 viability dye violet Invitrogen Cat NoH3569
Anti-ATRX antibody (ab97508) – Abcam
Anti-ATRX antibody Santa Cruz H300 sc-15408
Runx3 (39301 Active motif/ ThermoFisher)
Runx3 (653604 BioLegend)
H3.3 (09-838, Millipore/Merck)
H3K27ac (ab4729, Lot: GR276932-1, Abcam)
H3K9me3 (ab8898, lot GR3244171-1, Abcam)
H3K4me3 (ab8580, lot: GR273043-1, abcam)
H3K4me1 (ab8895, lot: GR283603-1, Abcam)
H3K27me3 (ab6002, lot: GR275911-6-1, Abcam)
H3K36me3 (ab9050, lot: GR233723-2, Abcam)
Recombinant Anti-GATA1 antibody [EPR17362] - ChIP Grade (ab181544, Abcam)
H3K4me3 (07-473, lot: 2664283, Millipore/Merc)
H3K4me1 (ab195391, lot: GR304893-2, Abcam)
sheep anti-DIG FITC (11207741910, Roche)
rabbit anti–sheep FITC (FI-6000, Vector Laboratories)

Validation

This antobody panel used in flow cytometry was setup using Fluorescence minus one (FMOs) and single stained controls on cultured erythroblasts throughout differentiation. This panel is used routinely to monitor erythroid differentiation in our ex-vivo culture system (Scott et al; 2020).

Anti-ATRX antibody (ab97508) – Abcam
Validated by Western blot and immunoprecipitation (https://www.abcam.com/atrx-antibody-ab97508.html?productWallTab=ShowAll)

Anti-ATRX antibody Santa Cruz H300 sc-15408
Checked by western blot and ChIP-qPCR. Used in published studies (Nguyen et al., 2017 and Voon et al., 2015)

Runx3 (39301 Active motif/ ThermoFisher)
"AML-2/Runx3 antibody tested by supershift. Detection of AML-2/Runx3 by EMSA analysis." (https://www.thermofisher.com/antibody/product/AML-2-Runx3-Antibody-Polyclonal/39301)

Runx3 (653604 BioLegend)
"Each lot of this antibody is quality control tested by ChIP Assay." (https://www.biolegend.com/fr-lu/products/go-chip-grade-purified-anti-runx3-antibody-14418)

H3.3 (09-838, Millipore/Merck)
"Use Anti-Histone H3.3 Antibody (Rabbit Polyclonal Antibody) validated in WB, ICC, DB, ChIP to detect Histone H3.3" (https://www.merckmillipore.com/FR/fr/product/Anti-Histone-H3.3-Antibody,MM_NF-09-838)

H3K27ac (ab4729, Lot: GR276932-1, Abcam)
Abpromise guarantee covers the use of ab4729 in the tested applications including ChIP.
(https://www.abcam.com/histone-h3-acetyl-k27-antibody-chip-grade-ab4729.html)

H3K9me3 (ab8898, lot GR3244171-1, Abcam)

"Histone H3 (tri methyl K9) antibody (ab8898) is specific for Histone H3 tri methyl Lysine 9. Shows slight cross-reactivity with tri methyl K27, which shares a similar epitope (please see Western blot image). Does not react with mono or di methylated K9."
Abpromise guarantee covers the use of ab8898 in the tested applications including ChIP.
(https://www.abcam.com/histone-h3-tri-methyl-k9-antibody-chip-grade-ab8898.html)

H3K4me3 (ab8580, lot: GR273043-1, abcam)
Abpromise guarantee covers the use of ab8580 in the tested applications including ChIP.
(https://www.abcam.com/histone-h3-tri-methyl-k4-antibody-chip-grade-ab8580.html)

H3K4me1 (ab8895, lot: GR283603-1, Abcam)
Abpromise guarantee covers the use of ab176877 in the tested applications including ChIP. 5 https://www.abcam.com/histone-h3-mono-methyl-k4-antibody-erp16597-chip-grade-ab176877.html)

H3K36me3 (ab9050, lot: GR233723-2, Abcam)
Abpromise guarantee covers the use of ab9050 in the tested applications including ChIP.
(https://www.abcam.com/histone-h3-tri-methyl-k36-antibody-chip-grade-ab9050.html)

Recombinant Anti-GATA1 antibody [EPR17362] - ChIP Grade (ab181544, Abcam)
Abpromise guarantee covers the use of ab181544 in the tested applications including ChIP.
(https://www.abcam.com/gata1-antibody-epr17362-chip-grade-ab181544.html).

H3K4me3 (07-473, lot: 2664283, Millipore/Merc)
"Anti-trimethyl-Histone H3 (Lys4) Antibody is a rabbit polyclonal antibody for detection of Histone H3 trimethylated at lysine 4. Also known as Anti-H3K4me3, this highly specific and well published antibody has been validated in ChIP, DB, WB, PIA, ChIP-seq." (https://www.merckmillipore.com/FR/fr/product/Anti-trimethyl-Histone-H3-Lys4-Antibody,MM_NF-07-473#)

H3K4me1 (ab195391, lot: GR304893-2, Abcam)
Abpromise guarantee covers the use of ab195391 in the tested applications including ChIP. (https://www.abcam.com/ab195391.pdf)

H3K27me3 (ab6002, lot: GR275911-6-1, Abcam)
Abpromise guarantee covers the use of ab6002 in the tested applications including ChIP (https://www.abcam.com/histone-h3-tri-methyl-k27-antibody-mabcam-6002-chip-grade-ab6002.html)

Eukaryotic cell lines

Policy information about cell lines

Cell line source(s)	CD34+ cells were extracted from patients with ATRX or unaffected donor or from leucocyte reduction filters obtained from the National Health Service Blood and Transplant (NHSBT). These cells were then expanded and differentiated in an ex-vivo culture system (Scott et al 2020)<br>Lymphoblastoid cell lines were derived from ATR-X cases or unaffected donors.<br>Fixed Drosophila melanogaster S2 cells were provided by the Milne group MRC Molecular Haematology Unit, MRC Weatherall Institute of Molecular Medicine, Radcliffe Department of Medicine, University of Oxford, Oxford, OX3 9DS, UK (Godfrey et al., 2019)
Authentication	During CD34+ differentiation, cells were staged using cytospins and FACS using a panel of 6 fluorophore-conjugated monoclonal antibodies (Scott et al 2020). LCLs derived from ATR-X patients were validated for the presence of ATRX mutation.
Mycoplasma contamination	CD34+ differentiation is a primary culture system, where cells are differentiated for 10 days so there are no mycoplasma issues.<br>LCLs were routinely tested for mycoplasma contamination.
Commonly misidentified lines<br>(See ICLAC register)	none

Human research participants

Policy information about studies involving human research participants

Population characteristics	Not relevant
Recruitment	ndividuals with ATR-X syndrome and their unaffected fathers were recruited for this study for lymphoblastoid studies. For erythroid studies in controls either healthy male volunteers were recruited or anonymous leucocyte reduction filters (obtained from the NHSBT) were used.
Ethics oversight	Scotland A Research Ethics Committee

Note that full information on the approval of the study protocol must also be provided in the manuscript.

ChIP-seq

Data deposition

- [x] Confirm that both raw and final processed data have been deposited in a public database such as GEO.
- [x] Confirm that you have deposited or provided access to graph files (e.g. BED files) for the called peaks.

Data access links
May remain private before publication.

https://www.ncbi.nlm.nih.gov/geo/query/acc.cgi?acc=GSE193038
https://www.ncbi.nlm.nih.gov/geo/query/acc.cgi?acc=GSE193310
https://www.ncbi.nlm.nih.gov/geo/query/acc.cgi?acc=GSE193311

Files in database submission

Ctr1_LCL_H3K9me3_norm_per100M.bw
Ctr2_LCL_H3K9me3_norm_per100M.bw
Ctr3_LCL_H3K9me3_norm_per100M.bw
Case1_LCL_H3K9me3_norm_per100M.bw
Case2_LCL_H3K9me3_norm_per100M.bw
Case3_LCL_H3K9me3_norm_per100M.bw
Ctr1_LCL_SC_input_norm_per100M.bw
Ctr2_LCL_SC_input_norm_per100M.bw
Ctr3_LCL_SC_input_norm_per100M.bw
Case1_LCL_SC_input_norm_per100M.bw
Case2_LCL_SC_input_norm_per100M.bw
Case3_LCL_SC_input_norm_per100M.bw
Ctr1_LCL_H3K4me3_norm_per100M.bw
Ctr2_LCL_H3K4me3_norm_per100M.bw
Ctr3_LCL_H3K4me3_norm_per100M.bw
Case1_LCL_H3K4me3_norm_per100M.bw
Case2_LCL_H3K4me3_norm_per100M.bw
Case3_LCL_H3K4me3_norm_per100M.bw
Ctr1_LCL_H3K4me1_norm_per100M.bw
Ctr2_LCL_H3K4me1_norm_per100M.bw
Ctr3_LCL_H3K4me1_norm_per100M.bw
Case1_LCL_H3K4me1_norm_per100M.bw
Case2_LCL_H3K4me1_norm_per100M.bw
Case3_LCL_H3K4me1_norm_per100M.bw
Ctr1_LCL_H3K36me3_norm_per100M.bw
Ctr2_LCL_H3K36me3_norm_per100M.bw
Ctr3_LCL_H3K36me3_norm_per100M.bw
Case1_LCL_H3K36me3_norm_per100M.bw
Case2_LCL_H3K36me3_norm_per100M.bw
Case3_LCL_H3K36me3_norm_per100M.bw
Ctr1_LCL_H33_norm_per100M.bw
Ctr2_LCL_H33_norm_per100M.bw
Ctr3_LCL_H33_norm_per100M.bw
Case1_LCL_H33_norm_per100M.bw
Case2_LCL_H33_norm_per100M.bw
Case3_LCL_H33_norm_per100M.bw
Ctr1_LCL_H3K27me3_norm_per100M.bw
Ctr2_LCL_H3K27me3_norm_per100M.bw
Ctr3_LCL_H3K27me3_norm_per100M.bw
Ctr1_LCL_H3K27ac_norm_per100M.bw
Ctr2_LCL_H3K27ac_norm_per100M.bw
Case1_LCL_H3K27ac_norm_per100M.bw
Case2_LCL_H3K27ac_norm_per100M.bw

Ctr1_LCL_H3K9me3_R1.fastq.gz
Ctr2_LCL_H3K9me3_R1.fastq.gz
Ctr3_LCL_H3K9me3_R1.fastq.gz
Case1_LCL_H3K9me3_R1.fastq.gz
Case2_LCL_H3K9me3_R1.fastq.gz
Case3_LCL_H3K9me3_R1.fastq.gz
Ctr1_LCL_SC_input_R1.fastq.gz
Ctr2_LCL_SC_input_R1.fastq.gz
Ctr3_LCL_SC_input_R1.fastq.gz
Case1_LCL_SC_input_R1.fastq.gz
Case2_LCL_SC_input_R1.fastq.gz
Case3_LCL_SC_input_R1.fastq.gz
Ctr1_LCL_H3K4me3_R1.fastq.gz
Ctr2_LCL_H3K4me3_R1.fastq.gz
Ctr3_LCL_H3K4me3_R1.fastq.gz
Case1_LCL_H3K4me3_R1.fastq.gz
Case2_LCL_H3K4me3_R1.fastq.gz
Case3_LCL_H3K4me3_R1.fastq.gz
Ctr1_LCL_H3K4me1_R1.fastq.gz
Ctr2_LCL_H3K4me1_R1.fastq.gz
Ctr3_LCL_H3K4me1_R1.fastq.gz
Case1_LCL_H3K4me1_R1.fastq.gz
Case2_LCL_H3K4me1_R1.fastq.gz
Case3_LCL_H3K4me1_R1.fastq.gz
Ctr1_LCL_H3K36me3_R1.fastq.gz
Ctr2_LCL_H3K36me3_R1.fastq.gz
Ctr3_LCL_H3K36me3_R1.fastq.gz
Case1_LCL_H3K36me3_R1.fastq.gz
Case2_LCL_H3K36me3_R1.fastq.gz
Case3_LCL_H3K36me3_R1.fastq.gz
Ctr1_LCL_H33_R1.fastq.gz
Ctr2_LCL_H33_R1.fastq.gz
Ctr3_LCL_H33_R1.fastq.gz
Case1_LCL_H33_R1.fastq.gz
Case2_LCL_H33_R1.fastq.gz
Case3_LCL_H33_R1.fastq.gz
Ctr1_LCL_H3K27me3_R1.fastq.gz
Ctr2_LCL_H3K27me3_R1.fastq.gz
Ctr3_LCL_H3K27me3_R1.fastq.gz
Ctr1_LCL_H3K27ac_R1.fastq.gz
Ctr2_LCL_H3K27ac_R1.fastq.gz
Case1_LCL_H3K27ac_R1.fastq.gz
Case2_LCL_H3K27ac_R1.fastq.gz
Ctr1_LCL_H3K9me3_R2.fastq.gz
Ctr2_LCL_H3K9me3_R2.fastq.gz
Ctr3_LCL_H3K9me3_R2.fastq.gz
Case1_LCL_H3K9me3_R2.fastq.gz
Case2_LCL_H3K9me3_R2.fastq.gz
Case3_LCL_H3K9me3_R2.fastq.gz
Ctr1_LCL_SC_input_R2.fastq.gz
Ctr2_LCL_SC_input_R2.fastq.gz
Ctr3_LCL_SC_input_R2.fastq.gz
Case1_LCL_SC_input_R2.fastq.gz
Case2_LCL_SC_input_R2.fastq.gz
Case3_LCL_SC_input_R2.fastq.gz
Ctr1_LCL_H3K4me3_R2.fastq.gz
Ctr2_LCL_H3K4me3_R2.fastq.gz
Ctr3_LCL_H3K4me3_R2.fastq.gz
Case1_LCL_H3K4me3_R2.fastq.gz
Case2_LCL_H3K4me3_R2.fastq.gz
Case3_LCL_H3K4me3_R2.fastq.gz
Ctr1_LCL_H3K4me1_R2.fastq.gz
Ctr2_LCL_H3K4me1_R2.fastq.gz
Ctr3_LCL_H3K4me1_R2.fastq.gz
Case1_LCL_H3K4me1_R2.fastq.gz
Case2_LCL_H3K4me1_R2.fastq.gz
Case3_LCL_H3K4me1_R2.fastq.gz
Ctr1_LCL_H3K36me3_R2.fastq.gz

Ctr2_LCL_H3K36me3_R2.fastq.gz
Ctr3_LCL_H3K36me3_R2.fastq.gz
Case1_LCL_H3K36me3_R2.fastq.gz
Case2_LCL_H3K36me3_R2.fastq.gz
Case3_LCL_H3K36me3_R2.fastq.gz
Ctr1_LCL_H33_R2.fastq.gz
Ctr2_LCL_H33_R2.fastq.gz
Ctr3_LCL_H33_R2.fastq.gz
Case1_LCL_H33_R2.fastq.gz
Case2_LCL_H33_R2.fastq.gz
Case3_LCL_H33_R2.fastq.gz
Ctr1_LCL_H3K27me3_R2.fastq.gz
Ctr2_LCL_H3K27me3_R2.fastq.gz
Ctr3_LCL_H3K27me3_R2.fastq.gz
Ctr1_LCL_H3K27ac_R2.fastq.gz
Ctr2_LCL_H3K27ac_R2.fastq.gz
Case1_LCL_H3K27ac_R2.fastq.gz
Case2_LCL_H3K27ac_R2.fastq.gz
Ctr1_LCL_ATRX_H300_norm_per100M.bw
Ctr2_LCL_ATRX_H300_per100M.bw
Ctr3_LCL_ATRX_H300_per100M.bw
Ctr1_LCL_ATRX_Abcam_per100M.bw
Ctr2_LCL_ATRX_Abcam_per100M.bw
Ctr3_LCL_ATRX_Abcam_per100M.bw
Ctr1_LCL_Input_DC_H300_per100M.bw
Ctr2_LCL_Input_DC_H300_per100M.bw
Ctr3_LCL_Input_DC_H300_per100M.bw
Ctr1_LCL_Input_DC_Abcam_per100M.bw
Ctr2_LCL_Input_DC_Abcam_per100M.bw
Ctr3_LCL_Input_DC_Abcam_per100M.bw
Ctr1_rep1_LCL_Runx3_Norm_per100M_under_peak.bw
Ctr1_rep2_LCL_Runx3_Norm_per100M_under_peak.bw
Case1_rep1_LCL_Runx3_Norm_per100M_under_peak.bw
Case1_rep2_LCL_Runx3_Norm_per100M_under_peak.bw
Ctr1_LCL_ATRX_H300_R1.fastq.gz
Ctr2_LCL_ATRX_H300_R1.fastq.gz
Ctr3_LCL_ATRX_H300_R1.fastq.gz
Ctr1_LCL_ATRX_Abcam_R1.fastq.gz
Ctr2_LCL_ATRX_Abcam_R1.fastq.gz
Ctr3_LCL_ATRX_Abcam_R1.fastq.gz
Ctr1_LCL_Input_DC_H300_R1.fastq.gz
Ctr2_LCL_Input_DC_H300_R1.fastq.gz
Ctr3_LCL_Input_DC_H300_R1.fastq.gz
Ctr1_LCL_Input_DC_Abcam_R1.fastq.gz
Ctr2_LCL_Input_DC_Abcam_R1.fastq.gz
Ctr3_LCL_Input_DC_Abcam_R1.fastq.gz
Ctr1_rep1_LCL_Runx3_R1.fastq.gz
Ctr1_rep2_LCL_Runx3_R1.fastq.gz
Case1_rep1_LCL_Runx3_R1.fastq.gz
Case1_rep2_LCL_Runx3_R1.fastq.gz
Ctr1_LCL_ATRX_H300_R2.fastq.gz
Ctr2_LCL_ATRX_H300_R2.fastq.gz
Ctr3_LCL_ATRX_H300_R2.fastq.gz
Ctr1_LCL_ATRX_Abcam_R2.fastq.gz
Ctr2_LCL_ATRX_Abcam_R2.fastq.gz
Ctr3_LCL_ATRX_Abcam_R2.fastq.gz
Ctr1_LCL_Input_DC_H300_R2.fastq.gz
Ctr2_LCL_Input_DC_H300_R2.fastq.gz
Ctr3_LCL_Input_DC_H300_R2.fastq.gz
Ctr1_LCL_Input_DC_Abcam_R2.fastq.gz
Ctr2_LCL_Input_DC_Abcam_R2.fastq.gz
Ctr3_LCL_Input_DC_Abcam_R2.fastq.gz
Ctr1_rep1_LCL_Runx3_R2.fastq.gz
Ctr1_rep2_LCL_Runx3_R2.fastq.gz
Case1_rep1_LCL_Runx3_R2.fastq.gz
Case1_rep2_LCL_Runx3_R2.fastq.gz
Ctr1_Ery_ATRX_norm_per100M.bw
Ctr12_Ery_ATRX_norm_per100M.bw

Ctr2_Ery_ATRX_norm_per100M.bw
Ctr4_Ery_ATRX_norm_per100M.bw
Ctr1_Ery_InputDC_norm_per100M.bw
Ctr2_Ery_InputDC_norm_per100M.bw
Ctr4_Ery_InputDC_norm_per100M.bw
Ctr2_Ery_GATA1.bw
Ctr3_Ery_GATA1.bw
Case1_Ery_GATA1.bw
Case2_Ery_GATA1.bw
Ery_ATRX_peaks.bed
Ery_GATA1_peaks.bed
LCL_ATRX_peaks.bed
LCL_runx3_peaks.bed
Ctr1_Ery_ATRX_R1.fastq.gz
Ctr12_Ery_ATRX_R1.fastq.gz
Ctr2_Ery_ATRX_R1.fastq.gz
Ctr4_Ery_ATRX_R1.fastq.gz
Ctr1_Ery_InputDC_R1.fastq.gz
Ctr2_Ery_InputDC_R1.fastq.gz
Ctr4_Ery_InputDC_R1.fastq.gz
Ctr2_Ery_GATA1_R1.fastq.gz
Ctr3_Ery_GATA1_R1.fastq.gz
Case1_Ery_GATA1_R1.fastq.gz
Case2_Ery_GATA1_R1.fastq.gz
Ctr1_Ery_ATRX_R2.fastq.gz
Ctr12_Ery_ATRX_R2.fastq.gz
Ctr2_Ery_ATRX_R2.fastq.gz
Ctr4_Ery_ATRX_R2.fastq.gz
Ctr1_Ery_InputDC_R2.fastq.gz
Ctr2_Ery_InputDC_R2.fastq.gz
Ctr4_Ery_InputDC_R2.fastq.gz
Ctr2_Ery_GATA1_R2.fastq.gz
Ctr3_Ery_GATA1_R2.fastq.gz
Case1_Ery_GATA1_R2.fastq.gz
Case2_Ery_GATA1_R2.fastq.gz
Ctr1_Ery_H33_norm_per100M.bw
Ctr4_Ery_H33_norm_per100M.bw
Ctr4_Ery_H3K27me3_norm_per100M.bw
Ctr1_Ery_H3K4me3_norm_per100M.bw
Ctr1_Ery_H3K4me1_norm_per100M.bw
Ctr1_Ery_InputSC_norm_per100M.bw
Ctr1_Ery_H3K27ac_norm_per100M.bw
Ctr2_Ery_H3K27ac_Rxnorm2.bw
Ctr3_Ery_H3K27ac_Rxnorm2.bw
Case1_Ery_H3K27ac_Rxnorm.bw
Case2_Ery_H3K27ac_Rxnorm2.bw
Ctr2_Ery_InputSC_filtered_pileup.bw
Ctr3_Ery_InputSC_filtered_pileup.bw
Case1_Ery_InputSC_filtered_pileup.bw
Case2_Ery_InputSC_filtered_pileup.bw
Ctr1_Ery_H33_R1.fastq.gz
Ctr4_Ery_H33_R1.fastq.gz
Ctr4_Ery_H3K27me3_R1.fastq.gz
Ctr1_Ery_H3K4me3_R1.fastq.gz
Ctr1_Ery_H3K4me1_R1.fastq.gz
Ctr1_Ery_InputSC_R1.fastq.gz
Ctr1_Ery_H3K27ac_R1.fastq.gz
Ctr2_Ery_H3K27ac_R1.fastq.gz
Ctr3_Ery_H3K27ac_R1.fastq.gz
Case1_Ery_H3K27ac_R1.fastq.gz
Case2_Ery_H3K27ac_R1.fastq.gz
Ctr2_Ery_InputSC_R1.fastq.gz
Ctr3_Ery_InputSC_R1.fastq.gz
Case1_Ery_InputSC_R1.fastq.gz
Case2_Ery_InputSC_R1.fastq.gz
Ctr1_Ery_H33_R2.fastq.gz
Ctr4_Ery_H33_R2.fastq.gz
Ctr4_Ery_H3K27me3_R2.fastq.gz

Ctr1_Ery_H3K4me3_R2.fastq.gz
Ctr1_Ery_H3K4me1_R2.fastq.gz
Ctr1_Ery_InputSC_R2.fastq.gz
Ctr1_Ery_H3K27ac_R2.fastq.gz
Ctr2_Ery_H3K27ac_R2.fastq.gz
Ctr3_Ery_H3K27ac_R2.fastq.gz
Case1_Ery_H3K27ac_R2.fastq.gz
Case2_Ery_H3K27ac_R2.fastq.gz
Ctr2_Ery_InputSC_R2.fastq.gz
Ctr3_Ery_InputSC_R2.fastq.gz
Case1_Ery_InputSC_R2.fastq.gz
Case2_Ery_InputSC_R2.fastq.gz

Genome browser session
(e.g. UCSC)

none

Methodology

Replicates

For ChIP-seq performed in LCLs: ATRX ChIP-seq: 6 replicates in total (from 3 different unaffected donors using 2 different antibodies). runx3 ChIP-seq: 2 replicates in total for the control (from the same unaffected donor (father of the ATR-X case 1) and 2 replicates in total for the case (from the same ATR-X case 1). H3K9me3, H3.3, H3K4me1 and H3K36me3 ChIP-seq: 3 replicates in total for the control (from 3 different unaffected donors) and 3 replicates in total for the case (from 3 different ATR-X cases). H3K27me3 ChIP-seq: 3 replicates in total for the control (from 3 different unaffected donors). H3K27ac ChIP-seq: 3 replicates in total for the control (from 2 different unaffected donors) and 3 replicates in total for the case (from 2 different ATR-X cases). H3K4me3 ChIP-seq: 4 replicates in total for the control (from 3 different unaffected donors) and 4 replicates in total for the case (from 3 different ATR-X cases).

For ChIP-seq performed in erythroblasts: ATRX ChIP-seq: 4 replicates in total (3 different unaffected donors: Ctr1_rep1_Ery_ATRX and Ctr1_rep2_Ery_ATRX are two replicates from the same donor). GATA1 ChIP-seq: 2 replicates in total for the control (from 2 different unaffected donors) and 2 replicates in total for the case (from 2 different ATR-X cases).
H3.3 ChIP-seq: 2 replicates in total for the control (from 2 different unaffected donors). H3K4me1 and H3K4me3 and H3K27me3 ChIP-seq: 1 replicate in total for the control. H3K27ac ChIP-seq: 3 replicates in total for the control (from 3 different unaffected donors) and 2 replicates in total for the case (from 2 different ATR-X cases).

More details in Supplemental Table 2.

Sequencing depth

All replicates have a sequencing depth > 10M reads. Paired-ends

Antibodies

Anti-ATRX antibody (ab97508) – Abcam
Anti-ATRX Santa Cruz H300 sc-15408
Runx3 (39301 Active motif/ ThermoFisher)
Runx3 (653604 BioLegend)
H3.3 (09-838, Millipore/Merck)
H3K27ac (ab4729, Lot: GR276932-1, Abcam)
H3K9me3 (ab8898, lot GR3244171-1, Abcam)
H3K4me3 (ab8580, lot: GR273043-1, abcam)
H3K4me1 (ab8895, lot: GR283603-1, Abcam)
H3K27me3 (ab6002, lot: GR275911-6-1, Abcam)
H3K36me3 (ab9050, lot: GR233723-2, Abcam)
Recombinant Anti-GATA1 antibody [EPR17362] - ChIP Grade (ab181544, Abcam)
H3K4me3 (07-473, lot: 2664283, Millipore/Merc)
H3K4me1 (ab195391, lot: GR304893-2, Abcam)

Peak calling parameters

In Erythroblast, ATRX samples were peak called using Homer findPeaks with the options -region -size 300 -minDist 1000 using their input as background control. For the final peakcall, the peaks from the four samples were merged using bedtools merge -d 200. The final peaks were filtered for selecting only peaks that were present at least in two samples. The final peaks were further filtered by removing peaks with a coverage under peak of ATRX < 35 and input > 70 to further reduced the number of artefacts based on visual inspection.
In LCLs, ATRX samples were peak called using Homer findPeaks with the options -region and
-size 150 -minDist 1000 (Ctr1_LCL_ATRX_H300 and Ctr1_LCL_ATRX_Abcam), -size 300 -minDist 1000 (Ctr3_LCL_ATRX_H300), -size 200 -minDist 1000 (Ctr2_LCL_ATRX_H300 and Ctr2_LCL_ATRX_Abcam) and -size 250 -minDist 1000 (Ctr3_LCL_ATRX_Abcam) using the input as background control. For the final peakcall, the peaks from each sample were merged using bedtools merge -d 200. The final peaks were filtered by removing peaks with a coverage under peak of ATRX < 35 and input >= 35 to reduce the number of artefacts based on visual inspection. The final peaks used for this study were further filtered for selecting only peaks that were present at least in three samples and independent of the antibody used.
GATA1 and runx3 ChIP-seq samples were peakcalled with macs2 callpeak defaults parameters using input as background control. The final peakcall was generated by merging (bedtools merge -d 100) the peak calls from the samples and filtered for selecting only peaks that were present at least in two samples.

Data quality	Quality of sequencing was verified by FastQC
Software	Software packages that were used for the analysis include FASTQC 0.11.9, Bowtie 2.3.2, Samtools 0.1.19, Bedtools 2.25.0 to 2.29.2, Deeptools 2.2.2 to 2.4.2, ucsctools 373, MACS2 (Zhang et al., 2008) and HOMER (Heinz et al., 2010). For ChIP-seq, data analysis was performed using an in-house pipeline: https://github.com/Hughes-Genome-Group/ NGseqBasic/releases.

Flow Cytometry

Plots

Confirm that:

- [x] The axis labels state the marker and fluorochrome used (e.g. CD4-FITC).
- [x] The axis scales are clearly visible. Include numbers along axes only for bottom left plot of group (a 'group' is an analysis of identical markers).
- [x] All plots are contour plots with outliers or pseudocolor plots.
- [x] A numerical value for number of cells or percentage (with statistics) is provided.

Methodology

Sample preparation	CD34+ human stem and progenitor cells were cultured according to Scott et al; 2021. On day 7 and 10 2x105 cultured erythroblasts were placed into FACS tubes , washed with FACS buffer and spun at 300g for 10 minutes. The supernatient was removed leaving 100ul and antibodies added and incubated for 30 min at 4oC in the dark. After this 2mls of FABS buffer was added and cells analysed using the AttuneNxT machine.
Instrument	AttuneNxT Acoustic Focusing Cytometer, Life Technology.
Software	FlowJo 10.0.8r1
Cell population abundance	This was FACS analysis the populations were not sorted.
Gating strategy	The cell population of interest was determined using FSC (between 250 and 650)K and SSC (100 and 500K) to exclude cell debris. Next the singlets were obtained by gating on the FSC-H v FSC-A. The live cells which were negative for the viability dye Hoechst 33258 where gated on and then analysed for expression of the erythroid cells surface markers specified in the antibody section above.

- [x] Tick this box to confirm that a figure exemplifying the gating strategy is provided in the Supplementary Information.
